# Efficacy of fusion imaging for immediate post‐ablation assessment of malignant liver neoplasms: A systematic review

**DOI:** 10.1002/cam4.6089

**Published:** 2023-05-16

**Authors:** Pragati Rai, Mohammed Yusuf Ansari, Mohammed Warfa, Hammad Al‐Hamar, Julien Abinahed, Ali Barah, Sarada Prasad Dakua, Shidin Balakrishnan

**Affiliations:** ^1^ Department of Surgery Hamad Medical Corporation Doha Qatar; ^2^ Department of Clinical Imaging Wake Forest Baptist Medical Center Winston‐Salem North Carolina USA; ^3^ Department of Clinical Imaging Hamad Medical Corporation Doha Qatar

**Keywords:** ablation techniques, ablative margin, image fusion, liver neoplasms, treatment outcomes

## Abstract

**Background:**

Percutaneous thermal ablation has become the preferred therapeutic treatment option for liver cancers that cannot be resected. Since ablative zone tissue changes over time, it becomes challenging to determine therapy effectiveness over an extended period. Thus, an immediate post‐procedural evaluation of the ablation zone is crucial, as it could influence the need for a second‐look treatment or follow‐up plan. Assessing treatment response immediately after ablation is essential to attain favorable outcomes. This study examines the efficacy of image fusion strategies immediately post‐ablation in liver neoplasms to determine therapeutic response.

**Methodology:**

A comprehensive systematic search using PRISMA methodology was conducted using EMBASE, MEDLINE (via PUBMED), and Cochrane Library Central Registry electronic databases to identify articles that assessed the immediate post‐ablation response in malignant hepatic tumors with fusion imaging (FI) systems. The data were retrieved on relevant clinical characteristics, including population demographics, pre‐intervention clinical history, lesion characteristics, and intervention type. For the outcome metrics, variables such as average fusion time, intervention metrics, technical success rate, ablative safety margin, supplementary ablation rate, technical efficacy rate, LTP rates, and reported complications were extracted.

**Results:**

Twenty‐two studies were included for review after fulfilling the study eligibility criteria. FI's immediate technical success rate ranged from 81.3% to 100% in 17/22 studies. In 16/22 studies, the ablative safety margin was assessed immediately after ablation. Supplementary ablation was performed in 9 studies following immediate evaluation by FI. In 15/22 studies, the technical effectiveness rates during the first follow‐up varied from 89.3% to 100%.

**Conclusion:**

Based on the studies included, we found that FI can accurately determine the immediate therapeutic response in liver cancer ablation image fusion and could be a feasible intraprocedural tool for determining short‐term post‐ablation outcomes in unresectable liver neoplasms. There are some technical challenges that limit the widespread adoption of FI techniques. Large‐scale randomized trials are warranted to improve on existing protocols. Future research should emphasize improving FI's technological capabilities and clinical applicability to a broader range of tumor types and ablation procedures.

## 1 INTRODUCTION

Liver neoplasms are a global health challenge worldwide, with high incidence and mortality rates.^1^ Hepatocellular carcinoma (HCC) is the most common type of primary liver cancer, originating from the hepatocytes, which are the main functional cells of the liver.^1^ It is a malignant tumor that often occurs in the context of chronic liver disease, such as cirrhosis or chronic hepatitis B or C infections. Other risk factors include alcohol abuse, non‐alcoholic fatty liver disease, and exposure to certain toxins like aflatoxin.^2^ Available treatment options include surgery, ablation therapies, embolization therapies, targeted drug therapies, immunotherapies, chemotherapy, and radiation therapy.^3^ The appropriate treatment option for an individual patient is a complex process that takes into consideration several factors such as staging of the cancer, liver function, tumor size, and location, as well as the patient's overall health.^4^ A multidisciplinary team of healthcare professionals, including hepatologists, oncologists, radiologists, and surgeons, are usually involved in making the decision.

Staging systems for hepatocellular carcinoma (HCC) play a crucial role in therapeutic decision‐making, as they help classify the extent of the disease and guide the selection of the most appropriate treatment options. By categorizing the tumor size, number, presence of vascular invasion, and the extent of liver function impairment, staging systems provide a framework for healthcare professionals to assess the prognosis and develop a tailored treatment plan for each patient.^4^

**FIGURE 1:**
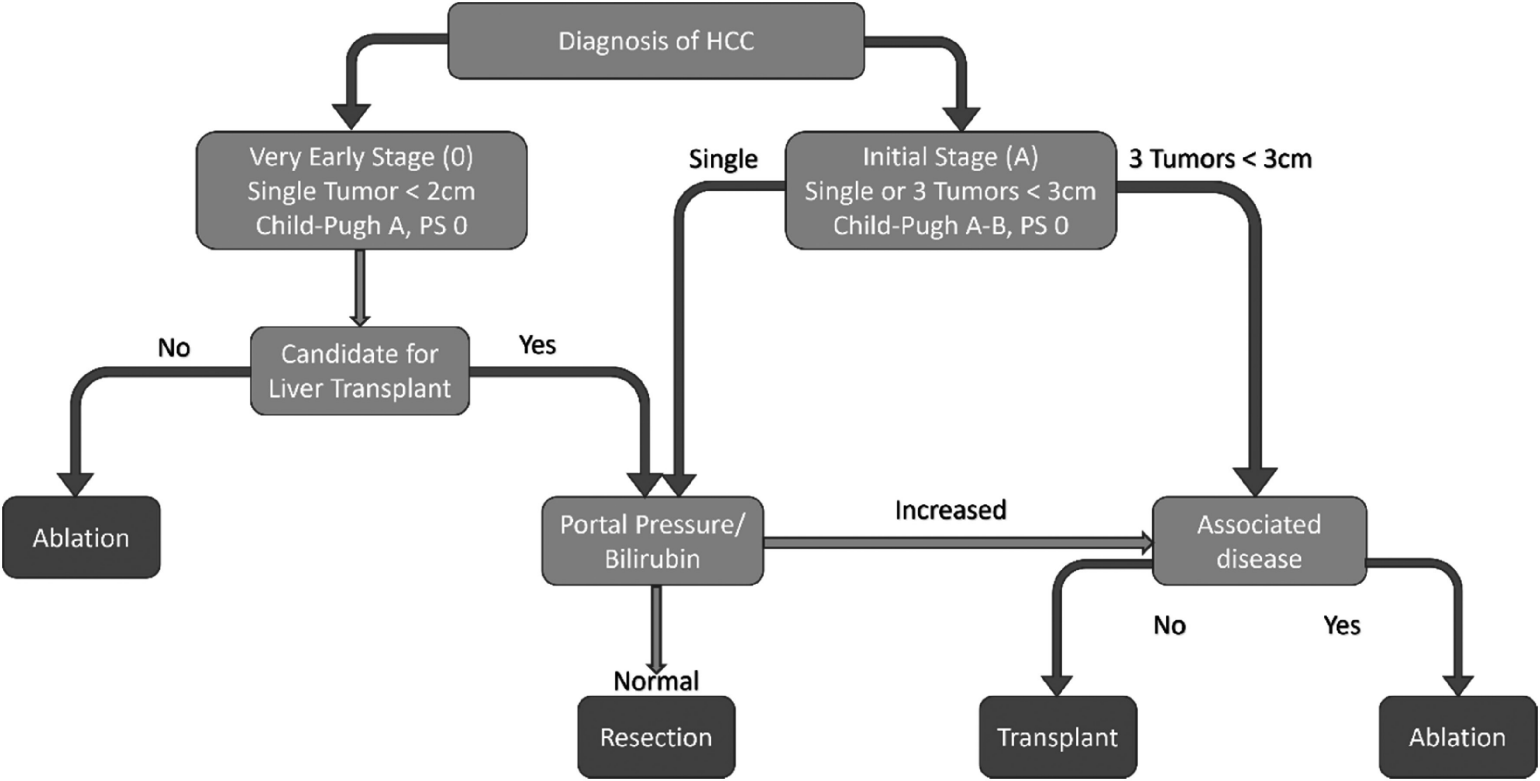
Staging classification and treatment algorithm for treating HCC based on BCLC criteria.

There are several staging systems for HCC, with the most widely used being the Barcelona clinic liver cancer (BCLC) staging system.^3^ Other systems include the American Joint Committee on Cancer (AJCC) TNM staging system, the Cancer of the Liver Italian Program (CLIP) score, and the Japan Integrated Staging (JIS) score.^5^ Each system has its strengths and limitations, and the choice of staging system may depend on regional preferences and clinical practice.^6^, ^7^, ^8^, ^9^ The BCLC staging system, for example, classifies HCC into five stages (0, A, B, C, and D) based on a holistic and comprehensive staging approach, which accounts for liver lesions, vascular invasion, liver dysfunction, and dissemination beyond the liver.^9^, ^10^ Due to this holistic approach, clinicians favor BCLC. Figure 1 above displays the BCLC algorithm and therapy suggestions.

For unresectable hepatic tumors, percutaneous thermal ablation has become a prominent therapeutic option.^11^ Compared to other therapeutic options mentioned above, patients who undergo thermal ablation (i.e., microwave ablation (MWA) and radiofrequency ablation (RFA)) generally experience lesser postoperative complications, infections, and shorter hospital stays.^12^ Even with these benefits, there are disadvantages and complications associated with thermal ablative therapeutic options, as shown in Table 1 below:

**TABLE 1:** Complications and disadvantages of thermal ablative therapy.

Thermal ablative therapy	Associated disadvantages	Associated complications
Radiofrequency ablation (RFA)	Limited effectiveness for larger tumors (typically over 3–4 cm) or tumors near major blood vessels. Heat sink effect: Cooling of the heated area by nearby blood flow can reduce the treatment's effectiveness.	Pain or discomfort at the treatment site. Bleeding or hematoma formation. Injury to adjacent organs or structures, such as bowel or bile ducts. Infection, although the risk is relatively low
Microwave ablation (MWA)	Similar to RFA, MWA may have limited effectiveness for larger tumors or those near major blood vessels. Less precise than RFA, which can increase the risk of damage to nearby structures.	

The effectiveness of ablative therapies for hepatocellular carcinoma (HCC) is evaluated based on a combination of several factors, which can include imaging studies, tumor response criteria (such as mRECIST), tumor marker levels (such as AFP), recurrence rates, and patient outcomes (including overall survival, progression‐free survival, and quality of life).^13^ In addition to the above, ablative therapy effectiveness is restricted by high rates of local tumor progression (LTP).^12^, ^14^, ^15^, ^16^ Insufficient safety margin, a large tumor diameter, a subcapsular position, closeness to blood vessels, and viable tumor cell adherence to the needle electrodes are some of the characteristics that contribute to high LTP rates.^17^ Thus, accurate assessment of posttherapeutic response is crucial in managing hepatic neoplasms and reduction of LTP in patients undergoing ablation therapy.^18^

Since the ablation zone cannot be evaluated histologically during the procedure, it is essential to assess the treatment outcome immediately after ablation to ascertain optimal procedural outcomes.^19^ Clinically, an effective treatment is said to be successful if no residual tumor or LTP is seen during the first and subsequent follow‐up visits. Ideally, the ablation zone has to cover the target tumor completely with a sufficient ablation margin (AM) for the procedure to be termed clinically successful.^20^ In general, an AM of 5–10 mm is recommended for clinically complete ablation.^17^ There is little evidence supporting these rather arbitrary values derived from surgical standards.^21^, ^22^

AM is a key predictor of LTP and therapeutic success, according to accumulating research.^23^, ^24^ Presently, the most frequent way of measuring AM in typical clinical settings is by comparing pre/post two‐dimensional (2D) scans and making manual measurements.^25^, ^26^ This method, however, is not accurate, cumbersome, and relies mainly on operator expertise. It is thus prone to error and subject to bias and can pose challenges even for experienced clinicians.^27^ Additionally, as more time passes after the ablation procedure, it becomes increasingly difficult to determine the treatment's effectiveness since tissue alterations in the ablative zone develop with time. Thus, an immediate post‐procedural evaluation of the ablation zone is crucial, as it could influence the need for a second‐look treatment or follow‐up plan.^28^

Image fusion (FI) strategies for the immediate assessment of the ablation zone have the potential to improve treatment outcomes by facilitating real‐time decision‐making, such as the need for supplementary ablation or adjustments to follow‐up plans.^29^ Image fusion involves combining two or more imaging modalities, such as computed tomography (CT), magnetic resonance imaging (MRI), and ultrasound (US), to create a single image with complementary information from each modality.^29^ Predicting treatment outcomes utilizing pre‐ and post‐ablation scans with image fusion methods has shown promising results recently due to the clinical significance of an instantaneous post‐procedural analysis.^11^, ^30^, ^31^ Novel imaging techniques have emerged in recent years that rely on image fusion to assess therapeutic outcomes, which can be visualized with the aid of mixed‐ and augmented reality platforms.^32^ Such an implementation enables clinicians to evaluate the therapeutic response immediately post‐ablation and carry out immediate retreatment, if necessary. This avoids the necessity for additional ablation procedures, thereby lowering the financial burden on patients.^33^

These emerging image technologies involve the fusion of different or same imaging modalities acquired before, during, and following percutaneous thermal ablation.^34^ Computed tomography (CT)‐CT, magnetic resonance (MR)‐MR, ultrasound (US)‐CT/MR, contrast‐enhanced ultrasound (CEUS)/US‐US/CEUS), and positron emission tomography (PET)/computed tomography (PET‐CT) are the commonly used FI techniques for assessing AM.^22^, ^35^, ^36^ Novel supportive software platforms enable early detection of the ablation zone and the extent of the AM. Commercial registration softwares are also available that merge pre‐ and post‐interventional images, enabling AM analysis and determination of immediate treatment effect.^11^, ^37^, ^38^ Quantitative AM analysis using image fusion has shown promising results in assessing treatment response immediately after ablation.^39^

**FIGURE 2:**
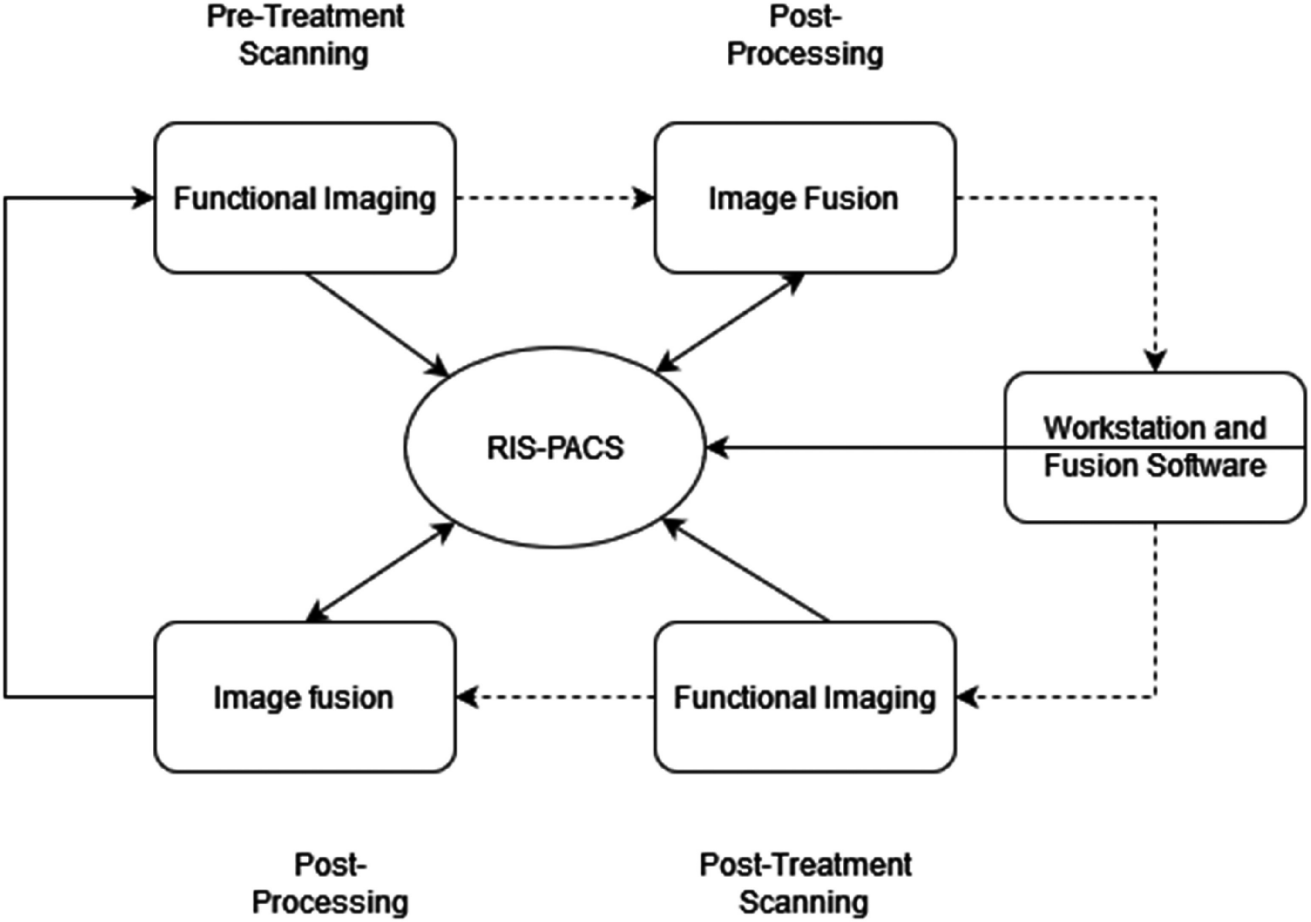
General workflow for patients undergoing fusion imaging (FI).

The general workflow used in FI involves acquiring baseline images, registering and fusing the images, validating the fused image, using the fused image for real‐time guidance during an interventional procedure, and evaluating the therapeutic response post‐procedure. This workflow can improve the accuracy and safety of interventional procedures in various clinical applications, including the management of hepatocellular carcinoma and other liver malignancies. The workflow for patients undergoing the FI procedure is depicted in Figure 2.

Systematic research on the viability and efficacy of FI systems for evaluating immediate post‐ablation response has not, to the best of our knowledge, been conducted. This study aims to focus on the clinical application of anatomical image fusion techniques for evaluating the immediate therapeutic response post thermal ablation in malignant liver neoplasms. While there have been studies on image fusion and its role in various aspects of thermal ablation for liver neoplasms, this review is unique in its focus on the viability and efficacy of fusion imaging (FI) systems for evaluating immediate post‐ablation response. This, coupled with the increasing popularity of these fusion imaging methods, has motivated us to examine existing publications in terms of their capabilities, accuracy, and limits of existing ablation confirmation. The goal of this study is to assess the efficacy of the image fusion techniques immediately post‐ablation in liver neoplasms.

## 2 MATERIALS AND METHODS

### 2.1 Study design

The systematic review was conducted in accordance with the preferred reporting items for systematic reviews and meta‐analyses (PRISMA) standards.^40^ PICO criteria were used to formulate the research question:

- (P) Population: primary or secondary liver neoplasms treated with thermal ablation [either of RFA, MWA, or srRFA].
- (I) Intervention: immediate evaluation of the posttherapeutic efficacy of liver ablation by fusion of pre‐and post‐ablation anatomical (CT or MRI or US or CEUS) images.
- (C) Comparator: none.
- (O) Outcome: evaluation of the technical efficacy of image fusion techniques in estimating immediate post‐ablation response

The protocol for this rapid review is registered with the PROSPERO (international prospective register for systematic reviews and meta‐analysis) with the registration number (CRD42021265980). The protocol has been previously described in detail.^41^

### 2.2 Eligibility criteria

The study design considered in the review included interventional (randomized and non‐randomized) and analytical observational studies [specifically controlled cohort studies (retrospective and prospective) and cross‐sectional studies and analytical case–control]. The search period was defined between January 2016 and June 2021. Studies explicitly assessing and reporting therapeutic response immediately post‐ablation using anatomical FI techniques in liver neoplasms were considered. Descriptive observational studies, narrative review articles, case series, case reports, commentaries, letters, opinions, and consensus statements were not considered. Liver tumors treated by methods other than MWA and RFA were not included in the final study. Articles published in non‐English languages were excluded.

### 2.3 Search strategy

EMBASE, PUBMED, and the Cochrane Library Central Registry electronic databases were used to perform a complete systematic search to discover studies investigating the image fusion procedures immediately after ablation in liver neoplasms. Following the evaluation of abstracts for relevance, full‐text papers were retrieved. There was a thorough screening of the reference list of chosen publications for other research that could be relevant. Both Medical Subject Headings (MeSH) and keyword searches were used to maximize the sensitivity of the search. The search terms “liver neoplasms” [MeSH Terms], “carcinoma, hepatocellular” [MeSH Terms], “ablation techniques” [MeSH Terms], “treatment outcome” [MeSH Terms] ‘ablative margin’, ‘fusion imaging’, ‘intraprocedural’, ‘three dimensional’ and ‘volumetric assessment’ were used in conjunction with the Boolean operators OR or AND.

### 2.4 Study selection

Covidence software was used to export the final records of the database search. Any duplicates were detected and removed by the software. Screening of the title and abstract of studies was independently carried out by three reviewers (MYA, PR, MW) based on the predefined eligibility criteria. Subsequent screening of the full text of selected articles was done to make further selections. The reasoning for articles that did not match the inclusion requirements for full texts was explained. The final list of articles was examined concurrently by the fourth reviewer (JA) against the predetermined qualifying criteria, and any disputes were addressed via discourse.

### 2.5 Data extraction

Three investigators (MYA, PR, MW) independently assessed and extracted the data from selected articles using a web‐based systematic review platform (Covidence).^42^ Relevant clinical characteristics include population demographics (number of patients, age, gender, geographic location), pre‐intervention clinical history (etiology of liver neoplasms, Child‐Pugh classification, and comorbidities), lesion characteristics (size), presence or absence of co‐interventions, and intervention type (RFA, MWA, sRFA) were extracted by the investigators. For the intervention type, variables including ablation type, fusion modality used, average fusion time, intervention metrics (for e.g., number of punctures, number of ablations), registration software used, technical success rate, ablative safety margin, supplementary ablation rate, technical efficacy rate, follow‐up duration, follow‐up imaging modality, LTP rates, and reported complications were documented. A fourth reviewer (JA) was consulted regarding any amendments to the extraction protocol.

### 2.6 Assessment of methodological quality

Two reviewers (HH, AB) utilized the Newcastle‐Ottawa scale to evaluate the methodological quality of the observational studies. Each study was evaluated based on three criteria: selection, comparability, and result.^43^ The Cochrane risk of bias 2.0 assessment instrument was used to quantify the risk of bias in randomized trials. This tool assesses the quality of studies in six domains. The risk for each domain is designated as low, unclear risk, and high risk of bias.^44^ Any discord or disagreements among the authors were addressed through dialog with the fourth reviewer (JA).

## 3 RESULTS

### 3.1 Literature search and study selection

**FIGURE 3:**
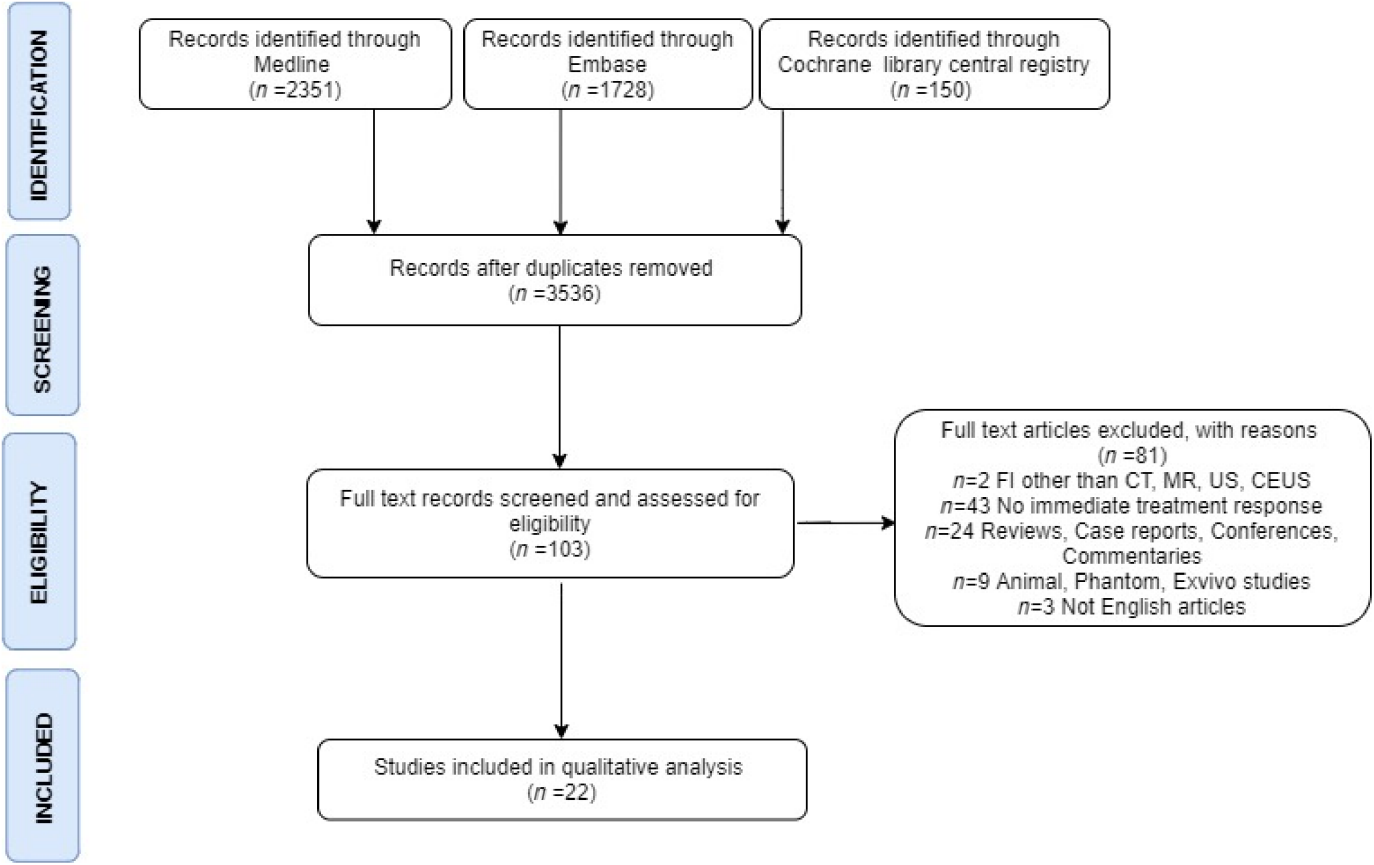
PRISMA flow diagram showing the selection of studies for systematic review.

We followed a priori the study protocol developed by Rai et al.^41^ The initial keyword search from the electronic databases yielded 4229 articles. Selected database search results were transferred to the Covidence software for further analysis, and 693 duplicates were eliminated. After reviewing the titles and abstracts of 3536 research, 3433 papers that did not match the inclusion criteria were omitted. The eligibility of the remaining 103 full‐text publications was then evaluated. Eighty‐one articles did not satisfy the qualifying requirements. In the end, 22 papers that matched the inclusion criteria were included in the qualitative analysis. The flowchart of the study selection process is tabulated in PRISMA format in Figure 3.

### 3.2 Study characteristics

The review comprises of 1 RCT, 10 non‐randomized prospective studies, and 11 retrospective observational studies. Of the 22 studies, 17 studies focused on HCC, 1 study included rare primary liver neoplasms, 2 studies focused on liver metastases, and 3 studies included both primary and secondary liver neoplasms. Nonetheless, most studies in the review focused on HCC. Study participants included 2735 patients diagnosed with HCC, 54 with liver metastasis, and 11 with other primary liver neoplasms. Most studies were conducted in Southeast Asia, including 13 in China, 6 in Japan, 1 in South Korea, and 2 in Europe. Across these studies, there were 2800 patients with 3648 liver tumors treated with thermal ablation. Table 2 and Table 5 provide a detailed breakdown of the patient population's demographics, illness history, and treatment parameters.

**TABLE 2:** Patient demographics from the studies included in the review.

Study ID	Country	Study design	Tumor type	Ablation type	Start date	End date	Age (years)	Sex (M/F)
Xu (2018)^45^	China	P	MLC	RFA/MFA	Aug‐15	Dec‐15	54 ± 11 (27–84)	94/21
Xu (2018)^46^	China	P	MLC	RFA/MFA	Aug‐15	Dec‐15	54 ± 11 (27–84)	61/15
Ma (2019)^33^	China	R	HCC	RFA/MFA	Jan‐08	Dec‐12	52.0 ± 10	CT/MR‐CEUS: 90/7 US: 70/13
Wang (2020)^47^	Japan	R	HCC	RFA	Jan‐15	Dec‐19	69.5 (37 ± 89)	90/25
Ding (2021)^48^	China	P	HCC	MWA	Jun‐20	Dec‐20	61.5 ± 8.0	18/06
Ju (2019)^49^	China	P	HCC	RFA/MWA	Dec‐10	Jul‐12	53.6 ± 10.4 (29–75)	91/7
Huang (2019)^50^	China	RCT	HCC	RFA	Jan‐16	Sep‐17	CT/MR‐CEUS: 52.7 ± 10. 3DUS‐CEUS: 54.8 ± 11.9	CT/MR‐CEUS: 110/14 3DUS‐CEUS: 111/14
Li (2016)^15^	China	P	HCC	RFA/MWA	Sep‐09	Jun‐12	53.7 ± 10.5 (29–75)	91/7
Zhang (2019)^51^	China	P	HCC	RFA	Oct‐16	Apr‐17	57.3 ± 11.0 (27–75)	71/13
Wu (2018)^52^	China	R	RLT	RFA/MWA	Jan‐14	Dec‐16	47 (28–64)	02/06
Makino (2016)^53^	Japan	R	HCC	RFA	Mar‐13	Jan‐ 14	76 (52 ± 89)	41/27
Yoon (2018)^11^	Korea	P	HCC	RFA	Oct‐10	Sep‐11	63.2 ± 9.5	55/13
Minami (2018)^54^	Japan	P	HCC	RFA/MWA	May‐14	Jul‐15	70 ± 12.0	39/14
Li (2017)^55^	China	P	HCC	RFA	Jan‐14	Apr‐14	NR	26/0
Ye (2019)^56^	China	P	HCC	RFA	Jan‐17	Apr‐17	56.0 ± 10.6 (26–75)	47/8
Xu (2021)^57^	China	R	HCC	RFA/MWA	May‐10	Oct‐16	53.2 ± 11.0 (25–84)	369/50
Sanga (2020)^58^	Japan	P	HCC	RFA	Jun‐17	Jun‐18	68 (44–88)	28/15
Long (2020)^59^	China	R	MLC	RFA/MFA	Oct‐10	Jun‐18	52 ± 11	441/61
Laimer (2020)^60^	Austria	R	HCC	srRFA	Jan‐09	Feb‐16	63.7 ± 10.2	90/20
Minami (2016)^61^	Japan	R	LM	RFA	Oct‐14	Mar‐16	60.9 ± 12.2 (41–89)	5/5
Laimer (2021)^30^	Europe	R	CLRM	srRFA	Jan‐09	Jan‐18	64.5 (31–87)	31/14
Minami (2020)^62^	Japan	R	HCC	RFA	May‐14	Nov‐16	71 ± 10.0	71/30

Abbreviations: CEUS, contrast‐enhanced ultrasound; CLRM, colorectal liver metastases; CT, computed tomography; HCC, hepatocellular carcinoma; LM, liver metastases; MFA, microwave ablation; MLC, malignant liver cancer; MRI, magnetic resonance imaging; P, prospective study; R, retrospective study; RCT, randomized clinical trials; RFA, radiofrequency ablation; RLT, rare liver tumor; US, ultrasound.

### 3.3 Assessment of methodological quality

The Newcastle Ottawa scale was used to evaluate the quality of 21 studies. There was a significant risk of bias in all the studies, with the average score being 4–6. On the other hand, risk of bias was performed on 1 RCT using the Cochrane Risk of Bias 2 tool. Except for concealment of allocation sequence and blinding participants and staff (performance bias), the bulk of domains were rated as having minimal bias risks. The results of the risk of bias assessment are presented in Table 3 and Table 4.

**TABLE 3:** Quality assessment of non‐randomized studies using Newcastle Ottawa scale.

STUDY	Selection				Comparability		Outcomes			Total Score
	Representatives of exposed cohort	Selection of external control	Ascertainment of exposure	Outcome of interest not present at the start of the study	Main factor	Additional factor	Assessment of outcomes	Sufficient follow‐up time	Adequacy of follow‐up	
Laimer et al.^23^	*		*				*	*	*	5
Laimer et al.^60^	*		*				*	*	*	5
Yoon et al.^11^	*		*	*			*	*	*	6
Xu et al.^50^	*	*	*				*	*	*	6
Xu et al.^38^	*	*	*	*			*			5
Xu et al.^39^	*		*	*			*			4
Long et al.^52^	*	*	*				*			4
Ju et al.^42^	*	*	*	*			*	*		6
Minami et al.^62^	*		*				*	*		4
Minami et al.^54^	*	*	*				*			4
Minami et al.^47^	*		*				*	*	*	5
Sanga et al.^51^	*		*	*			*	*	*	6
Wang et al.^40^	*		*				*	*	*	5
Makino et al.^46^	*	*	*				*	*	*	6
Ye et al.^49^	*		*	*			*	*		5
Zhang et al.^44^	*		*	*			*	*	*	6
Wu et al.^45^	*		*				*	*	*	5
Li et al.^48^	*		*	*			*			4
Li et al.^10^	*		*	*			*			4
Ding et al.^41^	*	*	*	*			*			5
Ma et al.^26^	*	*	*				*	*	*	6

**TABLE 4:** Quality assessment of randomized studies using Cochrane ROB‐2 tool.

Study	Random sequence generation	Allocation concealment	Blinding of participants and personal	Blinding of outcome assessment	Incomplete outcome data	Selective reporting	Overall bias
Huang et al.^50^	⊕	⊖	⊖	⊕	⊕	⊕	⊖

*Note*: ⊕: low risk, ⊖: high risk.

### 3.4 Clinical characteristics

#### 3.4.1 Intervention

For the intervention, the patients underwent RFA in ten studies, MWA in one study, SrRFA in two studies, and either MWA or RFA in nine studies. Co‐interventions (Trans arterial chemoembolization, TACE; percutaneous ethanol injection; PEI, etc.) were carried out in twelve studies.

#### 3.4.2 Imaging modality

Two studies examined CT fusion, and one study explored MR‐CT fusion, nine studies investigated CT/MR‐US/CEUS FI, eight studies examined US/CEUS‐US/CEUS fusion, and two studies investigated both US‐CEUS and CT/MRI‐CEUS in assessing the immediate post‐ablation response. There are three studies with US‐US overlay fusion,^53^, ^54^, ^61^ four with 3D US‐CEUS FI,^45^, ^46^, ^52^, ^58^ and two with 3D‐CEUS FI^48^, ^56^ focusing on the immediate therapeutic evaluation post‐ablation. The feasibility and efficacy of CT/MRI‐CEUS FI have been compared with CEUS or US in four studies^33^, ^45^, ^49^, ^50^ and CT/MRI‐CEUS or CEUS FI with CT–CT/MR‐MR in four studies.^48^, ^53^, ^55^, ^56^ Details of the imaging modality characteristics for each study are shown in Table 5.

**TABLE 5:** Clinical characteristics of patients included in the review.

Study ID	Patients/no. lesions	Tumor size (cm)	Co‐interventions	Clinical disease history
Xu (2018)^45^	115/157	1.9 ± 0.8	Y	Child‐Pugh classification (A/B/C): 106/9/0; Liver cirrhosis (Yes/No): 88/27, Alpha‐fetoprotein (200 μg/<200 μg): 90/25; Hepatitis virus (Negative/Positive): 30/85 Diagnosis (HCC/Intrahepatic cholangiocarcinoma/Colorectal liver metastases): 101/1/13 Combined surgeries or procedures: Artificial hydrothorax or ascites: 47 liver cancers in 37 patients Open surgery: 14 liver cancers in 10 patients; Laparoscopy: 8 liver cancers in 5 patients Procedure: RFA, MWA
Xu (2018)^46^	76/95	2.1 ± 0.9	Y	Hepatitis (Positive/negative): 63/13; Hepatic cirrhosis (Yes/no): 58/18; Child‐Pugh (A/B): 67/9, AFP (<200 μg/ ≥200 μg): 59/17 Diagnosis/Disease (HCC/MLC/ICC): 66/9/1 Surgeries (Artificial ascites/artificial pleural effusion/open operation/laparoscopy): 18/6/9/4 Procedure: RFA, MWA
Ma (2019)^33^	195/219	CT/MR‐CEUS: 1.8 (1.0–4.2) US: 2.0 (1.0–4.0)	Y	HBsAg (+/−): 95/2, 81/2; HCV Ab (+/−): 1/96, 0/83; Liver cirrhosis (yes/no): 87/10, 73/10 AFP (<20 ng/mL/≥20 ng/mL): 57/40, 40/4; Child‐Pugh (A/B): 86/11, 76/7 Diagnosis: HCC Procedure (RFA/MWA): 96/14, 53/37; Combined use of TACE or PEI (yes/no): 29/81, 34/56 TACE/PEI/TACE+PEI: 22/4/3, 28/6/0; Artificial ascites (yes/no): 26/84, 27/63 Number of punctures†: 2 (1,6), 1 (1,5); Number of ablations†: 2 (1,6), 1 (1,5) (†: Median (min, max))
Wang (2020)^47^	115/115	1.59 ± 0.46	N	Child‐Pugh (A/B): 106/9; HCV/HBV/others: 71/15/29; Location of lesion (right/left): 71/44 AFP: 132.2 (2.0–2885.0) Diagnosis: HCC Procedure: RFA
Ding (2021)^48^	24/32	2.3 ± 0.6	N	Etiology of liver disease: Hepatitis B virus: 20; Hepatitis C virus: 2: Alcohol: 1; Autoimmune hepatitis: 1; cirrhosis: 23 Diagnosis: HCC Procedure: MWA
Ju (2019)^49^	190/246	1.96 ± 0.77	Y	HBV or HCV (yes/no): 97/1, 90/2,1; Liver cirrhosis (yes/no): 82/16, 79/1 Child‐Pugh classification (A/B): 89/9, 77/15; AFP (<200 ng/mL/> = 200 ng/mL): 88/10, 76/16 Diagnosis: History of HCC treatment (yes/no): 54/72, 62/58 Treatment procedure: Ablation method (RFA/MWA): 94/4, 90/2 TACE (yes/no): 41/85, 44/76
Huang (2019)^50^	374/456	CT/MR‐CEUS: 1.88 ± 0.8 3DUS‐CEUS: 1.83 ± 0.6	Y	Liver cirrhosis (Yes/No): 108/16, 110/15, 107/18; Child‐Pugh class (A/B): 122/2, 123/2, 121/4 ALBI grade (1/2/3): 85/37/2, 83/41/1, 83/42/0 AFP level (<=200 ng/mL /> = 200 ng/mL): 107/17, 107/18, 103/22 Diagnosis: HCC Treatment procedure: Ablation method (RFA/MWA): 112/41, 107/46, 108/42 Associated with other procedures# (Yes/No): 44/109, 42/111, 44/106
Li (2016)^15^	98/126	1.96 ± 0.7	Y	Viral hepatitis/alcoholic liver disease: 97/1; Liver cirrhosis (yes/no): 82/16 Child‐Pugh class (A/B): 89/9; AFP (ng/mL) (<20/≥20): 65/33 Diagnosis: History of HCC (yes/no): 50/48 Procedure: RFA plus TACE (yes/no): 41/85
Zhang (2019)^51^	84/84	2.1 ± 0.7	N	Hepatitis B virus (+/−): 78/6; Hepatitis C virus (+/−): 3/81; AFP (<=20 μg/L/>20 μg/L): 41/43 Serum total bilirubin (mmol/L): 15.8 ± 10.7 (5.7–92.2); Serum ALT level (U/L): 39.0 ± 31.2 (6–187) Child‐Pugh Class (A/B): 81/3 Diagnosis: HCC Procedure: RFA
Wu (2018)^52^	08/12	1.45 (0.6–2.5)	Y	Pathology: Inflammatory myofibroblastic tumor, Inflammatory myofibroblastic tumor, Metastasis of spleen inflammatory myofibroblastic tumor, B‐cell‐derived lymphoma or dendritic cell tumor, EBV‐associated lymphoepithelioma‐like cholangiocarcinoma Diagnosis: rare liver tumor diseases Procedure: RFA and MWA
Makino (2016)^53^	68/85	1.19	N	Etiology (HBV/HCV/others): 9/52/7 Diagnosis: HCC Number of ablations per tumor at the first treatment session: 2 (1–5) † Number of treatment sessions per tumor before complete ablation: (1/2/3) 80/4/1 Follow‐up period (months): 17 (3–27) † (†: Median (min, max)) Procedure: RFA
Yoon (2018)^11^	68/88	1.6 ± 0.6	Y	Presence of underlying liver disease (%): Chronic hepatitis B: 79.4 (54/68); Chronic hepatitis C: 8.8 (6/68); Alcoholic liver disease: 4.4 (3/68) Non‐B non‐C liver cirrhosis: 5.9 (4/68); Primary biliary cirrhosis: 1.5 (1/68); Child‐Pugh classification: (%) Child‐Pugh A: 95.6 (65/68); Child‐Pugh B: 4.4 (3/68) History of local treatment (%): RFA: 8.8 (6/68); TACE: 22.1 (15/68); PEI: 4.4 (3/68); PEI and TACE: 5.9 (4/68); RFA and TACE: 4.4 (3/68) Diagnosis: HCC Procedure: RFA
Minami (2018)^54^	53/68	1.8 ± 0.7	N	Etiological cause of HCC: Hepatitis B: 4 (7.5); Hepatitis C: 34 (64.2); non‐B non‐C: 15 (28.3); Mean serum albumin level (g/dl)*: 3.7 ± 0.6; Mean serum total bilirubin level (g/dl)*: 1.0 ± 1.0 Child‐Pugh class (A/B): 43 (81.1)/ 10 (18.9) Serum AFP level (< 20 ng/mL / < 20 ng/mL / 20–200 ng/mL): 33 (62.2)/ 19 (35.8)/ 1 (1.9) Diagnosis: HCC Procedure: RFA
Li (2017)^55^	26/30	1.9 ± 6.5	N	Virus hepatitis/alcoholic liver disease/no diffuse hepatic disease: 26/0/0 Diagnosis: HCC Procedure: RFA
Ye (2019)^56^	55/55	2.0 ± 0.7	N	Hepatitis B virus (+/−): 50/5; Hepatitis C virus (+/−): 2/53; AFP (<=20 μg/L/>20 μg/L): 34/21 Serum albumin (g/L): 38.9 ± 3.8 (29.8–48.0); Serum total bilirubin (lmol/L): 15.4 ± 7.5 (6.2–41.6) Serum ALT level (U/L): 35.1 ± 25.6 (9–161); Prothrombin time (s): 12.8 ± 1.2 (10.7–17.1) Child‐Pugh class A/B: 54/1 Diagnosis: HCC Procedure: RFA
Xu (2021)^57^	440/543	1.8 (1.0–4.9)	Y	Liver cirrhosis (yes/no): 356 (85.0%)/63 (15.0%) AFP (<20/20–200/>200): 234 (55.9%)/112 (26.7%)/73 (17.4%) Etiology of liver disease (HBV/HCV/alcoholic/none): 397 (94.7%)/12 (2.9%)/2 (0.5%)/8 (1.9%) Child‐Pugh (A/B): 388 (92.6%)/31(7.4%) Diagnosis: HCC Previous therapy for HCCs (none/OP/TACE/RFA/MWA/OP +TACE/OP + RFA/OP + MWA/RFA + TACE/MWA + TACE/OP + RFA + TACE): 272 (64.9%)/67 (16.0%)/10 (2.4%)/23 (5.5%)/3 (0.7%)/ 11 (2.6%)/7 (1.7%)/2 (0.5%)/15 (3.6%)/2 (0.5%)/7 (1.7%) Procedure: Thermal ablation (RFA/MWA) 312 (74.5%)/107 (25.5%) Combined therapy (none/TACE) 364 (86.9%)/55 (13.1%) None/artificial pleural fluid/artificial ascites/artificial pleural fluid + artificial ascites/ laparoscopic surgery (laparoscopic cholecystectomy)/cholecystectomy: 296 (70.6%)/16 (3.8%)/97 (23.1%)/4 (1.0%)/9 (2.1%)/3 (0.7%)
Sanga (2020)^58^	43/50	1.53 (0.8–2.9)	N	Etiology of HCC: Hepatitis B/hepatitis C/others: 11/26/6; Child‐Pugh classification (A/B): 40/3 Diagnosis: HCC Procedure: RFA
Long (2020)^59^	502/805	1.66	Y	Hepatitis virus infection (Yes/No): 473 (94.2%)/29 (5.8%); Cirrhosis (Yes/No): 416 (82.9%)/86 (17.1%) Cause of cirrhosis (HBV/HCV/HBV + HCV/alcohol): 484 (96.4%)/15 (3.0%)/1 (0.2%)/1 (0.2%)/1 (0.2%) AFP (<200/≥200): 420 (83.7%)/82 (16.3%); Child Pugh (A/B): 477 (95.0%)/25 (5.0%) Diagnosis: HCC or other malignancies Procedure: RFA/MWA
Laimer (2020)^60^	110/176	2.52 ± 1.49	N	Cirrhosis, *n* (%): 95 (86.4); HBV: 6 (5.5); HCV: 19 (17.3) Fatty liver disease (AFLD and NAFLD): 45 (40.9) Other 25: (27.7) Child‐Pugh (A/B/C): 78 (82.1%)/ 15 (15.8%)/ 2 (2.1%); AFP (ng/l): 7 (3.5–24.6) Procedure: stereotactic radiofrequency ablation (SRFA)
Minami (2016)^61^	10/12	1.6 ± 0.9	N	Secondary hepatic malignancies included patients with colorectal cancer (*n* = 4); breast cancer (*n* = 2), lung cancer (*n* = 1); gastrointestinal stromal tumor (*n* = 1); pancreatic neuroendocrine tumor (*n* = 1); and adrenocortical carcinoma (*n* = 1) Diagnosis: Liver metastasis Procedure: RFA
Laimer (2021)^30^	45/76	2.42 (0.3–7.5)	Y	Diagnosis: LM Procedure: SRFA (baseline characteristics not provided in a table)

Abbreviations: RFA: radiofrequency ablation; MWA: microwave ablation, SRFA: stereotactic radiofrequency ablation, HCC: hepatocellular carcinoma, MLC: malignant liver carcinomas, FI: fusion imaging, LTP: local tumor progression, US: ultrasound, MRI: magnetic resonance imaging, CEUS: contrast‐enhanced ultrasound, CT: computed tomography, AM: ablation margin, Y: yes, N: no, AFP: alpha‐fetoprotein, HBV: hepatitis B virus, HCV: hepatitis C virus, LM: liver metastasis, TACE: transarterial chemoembolization, ALT: alanine transaminase, HBsAg: hepatitis B surface antigen, ICC: intrahepatic cholangiocarcinoma, HCC: hepatocellular carcinoma.

#### 3.4.3 Follow‐up

Nineteen of the 22 studies reported the follow‐up duration for their studies. These studies included follow‐up periods ranging from 1 to 66 months.

#### 3.4.4 Equipment

Most of the studies used software such as Virtual Navigator, Volume Navigation, Percunav, and Hepacare. The machines used widely varied, with some commonly used ones being Mylab Twice^63^ and/or Mylab90. Other machines used in the studies include Logiq E9, 3D workstation, and Mindray.^64^ The details of equipment are present in Table 6.

**TABLE 6:** Comparison of outcomes across the different studies included in the review.

Study ID	Fusion imaging modality	Equipment	Software employed for FI	Safety margin achieved	Duration of follow‐up (months)	LTP (%)	Applicable rate (%) and/or Technical success (%) and/or Registration time	Complications	Efficacy and/or Supplementary ablation rate
Xu (2018)^45^	CT/MR‐CEUS, US‐CEUS	MyLab 90/ MyLab Twice	Virtual Navigator	NR	3	NR	US‐CEUS: Applicable rate: 61.1% (96/157) Registration rate: 93.8% (90/96) CT/MRI‐CEUS: Applicable rate: 98.7% (155/157) Registration rate: 81.3% (126/155) Registration time CT/MR‐CEUS: 5.7 ± 2.7 min US‐CEUS: 3.7 ± 1.7 min	No major complications	Total of 150 tumors achievedcomplete ablation, yielding a technical efficacy rate of 99.3% (150/151) Supplementary ablation rate: 24.80%
Xu (2018)^46^	3DUS‐CEUS	MyLab Twice	Virtual Navigator	92.13%	NR	NR	3D US‐CEUS: Registration success rate: 93.7% (89/95) Registration time: 4.0 ± 1.1 min.	No major complications	Effectiveness rate: 98.8% (81/82) Supplementary ablation rate: 33.70%
Ma (2019)^33^	CT/MR‐CEUS	MyLab 90/ MyLab Twice	Virtual Navigator	60%	66 (1–72)	6.3	CT/MR‐CEUS FI: Whole technical efficacy rate: 100% (110/110) Technical efficacy rate of difficult lesion: 100% (64/64) US: Whole technical efficacy rate: 86.7% (78/90) Technical efficacy rate of difficult lesion: 84.6% (44/52) Registration time: 5–10 mins	All major complications of the thermal ablation were recorded	Technical efficacy of the FI group was higher than in the US group (100% vs. 86.7%, *p* < 0.001) Supplementary ablation rate: 14.50%
Wang (2020)^47^	EOB‐MR/US	LOGIQ E9/E10 unit	V Nav System Global positioning system (GPS)	NR	376.7 (21–1329) days	14.8%	NR	NR	NR
Ding (2021)^48^	3DCEUS FI 3DCECT (1 week post‐ablation)	Philips EPIQ 7	PercuNav	39.00%	1	NR	3D CEUS: fusion success rate: 95.8% (23/24) time: (4.1 ± 1.8) min Enhanced CT: Success rate: 100% (24/24) Time: 3–8 (4.6 ± 1.5) min	NR	NR
Ju (2019)^49^	CT/MR‐CEUS, 3DUS‐CEUS	MyLab Twice	Virtual Navigator	65.9	44 (1–75)	8.7	Technical success rate: CEUS‐CT/MR fusion: 86.3% (126/146) CEUS: 98.4% (120/122) Registration time: 4.9 ± 2.0	Major complications related to ablation	Efficacy rate: 99.2% (125/126) and 94.2% (113/120) Supplementary ablation rate: 14.3% (18/126) and 4.2% (5/120)
Huang (2019)^50^	CT/MR‐CEUS, 3DUS‐CEUS	Mylab Twice	Virtual navigator, 3D software		24 (1–37)	CT/MR‐CEUS:4.1 3DUS‐CEUS: 4.1	applicable rates: CT/MR‐CEUS: 94.8% (145/153) 3DUS‐CEUS: 94.1% (144/153) CEUS: 96.0% (144/150) Technical Success rate: CT/MR‐CEUS:100 3DUS‐CEUS: 100 Ablation time: CT/MR‐CEUS: 56 (20–183) min 3DUS‐CEUS: 55 (10–179) min CEUS:: 53 (10–165) min	Major complications related to ablation rate of major complications: CT/MR‐CEUS: 0.84% (1/119) 3DUS‐CEUS: 0.85% (1/118) CEUS: 0.83% (1/121)	Efficacy rate: CT/MR‐CEUS: 99.3% (144/145) 3DUS‐CEUS: 100% (144/144) CEUS: 100% (144/144) Supplementary ablation rate: CT/MR‐CEUS: 13.8% (20/145) 3DUS‐CEUS: 20.1% (29/144) CEUS: 9.0% (13/144)
Li (2016)^15^	CEUS‐CT/MR	MyLab Twice	Virtual Navigator	65.80%	25 (4–37)	4.80%	Fusion success rate: 96.2% (126/131) Time: 4.9 ± 2.0 (3–13)	Ablation complications recorded	Efficacy rate: 99.20% Supplementary ablation rate: 21.80%
Zhang (2019)^51^	3DCEUS‐CEUS	Mindray DC8	Fusion	36.40%	9.6 (4.9 ± 16.6)	10.40% (8/77 during the follow‐up period)	automatic registration: 57.1% (48/84), time: 4–12 sec interactive registration: 91.7% (77/84), time: 4.2 ± 1.8 min	NR	NR
Wu (2018)^52^	CT/MR‐CEUS, 3DUS‐CEUS	MyLabTwice/MyLabClassC	Virtual Navigator	NR	13 (2–32)	0%	Ablation success rate: 100%	NR	100% (12/12) success rate of all nodules
Makino (2016)^53^	CT/MR‐US Overlay fusion	LOGIQ E9	Volume navigation	91.10%	NR	8.9%	Ablation results for CT/MR with CEUS: Margin (0–5 mm): 73 (92.4%) Margin (>5 mm): 6 (7.6%) (Treatment evaluation was impossible using CEUS in six HCCs because the tumors were located far belowthe body surface) Registration time: <10 mins	NR	Efficacy: (77/79)
Yoon (2018)^11^	MR‐CT	3D workstation (Leonardo)	Hepacare, Siemens Healthcare	80.7%	48.0 (0.9 ± 72.6)	30.30% (20/66)	sufficient ablative margins: software: 80.7% (71/88) Visual: 84.1% (74/88)	NR	Efficacy: 98.5% (67/68 patients)
Minami (2018)^54^	US‐US overlay	LOGIQ E9	Volume navigation	86.80%	17.8 ± 7.6	0%	Technically effective ablation: 59 (86.8%)	Two patients demonstrated distantsingle metastases in the liver. All procedures were performedsuccessfully without immediate or late complications.	US‐US image overlay correctly predictedearly CT evaluation with an accuracyof 92.6% (63/68)
Li (2017)^55^	CEUS‐CT/MR	MyLab Twice	Virtual Navigator	50%	NR	NR	sensitivity, specificity, and accuracy for: US‐CT image fusion: 93.8, 85.7, and 91.3% CEUS‐CT/MR imaging fusion: 100.0, 80.0, and 90.0%, Registration time: 8.5 ± 2.8 (4–12)	NR	Clinical success is 100% for CEUS‐CT/MR and CT–CT/MR‐MR fusion
Ye (2019)^56^	3DCEUS‐3DCEUS	Mindray, Shenzhen (US) (IQQA)‐ CT fusion	(IQQA)‐ CT fusion, Virtual Place Advance Plus(US)	NR	13.6 (5.6–18.)	9.4%	3DCEUS fusion registration success rate: 96.4% (53/55) CT Fusion registration success rate: 98.2% (54/55) Registration time: 3DCEUS fusion: 4.4 ± 1.9 min CT Fusion: 2 ± 9 min	NR	AM evaluation was successful both on 3DCEUS and CT fusionimaging in 53 (96.4%) tumors.
Xu 2021^57^	CT‐MR/US	MyLab 90/ MyLab Twice	Virtual Navigator	97%	31 (6 ± 80)	Cumulative LTP rates: 3.2%, 5.6% and 7.2% at 1, 3, and 5 years	CT/MR‐US fusion imaging was successfully registered in 419 patients with 502 nodules. Registration time: 5.0 (1.3–18) min	Major complication ratewas 1.9%.	Technique efficacy rateof thermal ablation was 99.4%. Supplementary ablation rate: 19.30%
Sanga (2020)^58^	US‐CEUS	LOGIQ E9	V Nav System: GPS marks	48%	21.4 (15 ± 27)	Local tumor progression in areas of thetumor adjacent to the portal vein (2%)	The registration success rate of CEUS/US fusion imaging was 100% (50/50). Registration time: < 2 mins	NR	Success rate: 33/50 HCCs in the first ablation Success rate: 16/17 HCCs in the second ablation Net success rate: 98% (49/50) the concordance rate for evaluations betweenintraprocedural CEUS/US fusion imaging and CECT/CEMRI performed one month after RFA was 88% (44/50). Supplementary ablation rate: 34%
Long (2020)^59^	CT/MR‐US	MyLab 90/MyLab Twice/ MyLab	Virtual Navigator	89.6%DC 95.9%NDC	30 (1–96)	local tumor progression rate: Difficult case group (1 year: 3.2%; 3 years: 7.6%; 5 years: 7.6%) and non‐difficult case group (1 year: 2.1%; 3 years: 5.5%; 5 years: 11.6%)	Successful registration: 502/517 patients	Major complication rate was 1.8% (11/608)	Technical efficacy rate: 99.4% (800/805) Supplementary ablation rate: 25.5% in DC and 16.7% In NDC
Laimer (2020)^60^	CT–CT	Siemens Healthineers	Syngo.via VB20A	NR	26.0 ± 10.3	8.20% (9/110)	Initial automatic registration was deemed unsatisfactory. Follow‐up manual correction by multiplanar slight shifting performed Registration time: >15 mins	NR	Minimum ablative margin (MAM) <5 mm: 62.5% (110) Minimum ablative margin (MAM) >5 mm: 37.5% (66) Median MAM = 3 mm (2‐7 mm)
Minami (2016)^61^	US‐US overlay	LOGIQ E9	Volume Navigation	NR	1–13.71	None	NR	There were no serious adverse events orprocedure‐related complications (e.g., hemorrhage, infection,hepatic failure or death). One patient showed distant liver metastasis	Ten liver metastases(83%) were depicted as defects with a clear margin, and 2nodules (17%) were depicted as defects with an unclearmargin.
Laimer (2021)^30^	CT–CT	Siemens SOMATOM Sensation Open	Ablation‐fit TM; R.A.W. Srl)	NR	36.1 ± 18.5	11.80%	Registration time: 10 (5–20) mins	NR	NR
Minami (2020)^62^	US‐US overlay	LOGIQ E9	Volume Navigation	89.3% (108/121)	1–29	0.8% (1/121)	Technical success rate: 100% (101/101) for the US‐US overlay fusion group	Overall incidence of serious adverse events (grade 3 or 4) and moderate adverse events (grade 2) was 1.4% (6/426) and 3.8% (16/426), respectively in intervention and control groups.	100% technical efficacy 5‐mm safety margins achieved in 89.3% (108/121) of nodules in the US‐US overlay fusion group, which was significantly higher than the 47.0% (213/453) achieved in the control group

Abbreviations: AM, ablation margin; CEUS, contrast‐enhanced ultrasound; CT, computed tomography; DC, difficult cases; FI, fusion imaging; HCC, hepatocellular carcinoma; LTP, local tumor progression; MLC, malignant Liver carcinomas; MRI, magnetic resonance imaging; MWA, microwave ablation; NDC, non‐difficult cases; NR, not reported; RFA, radiofrequency ablation; srRFA, stereotactic radiofrequency ablation; US, ultrasound.

### 3.5 Outcomes metrics reported

Within the context of FI procedures for liver cancer treatment, metrics in published literature used to describe different aspects of the procedure's effectiveness include applicable rate, technical success rate, registration success rate, fusion time, ablative margin, and safety margin achieved, supplementary ablation carried out, LTP rate, and post‐procedural complications. These metrics provide insights into various aspects of the procedure, from the efficiency of the imaging process to the effectiveness of the treatment and potential complications.

*Applicable rate* refers to the proportion of cases in which the fusion imaging technique can be successfully applied or utilized for a specific purpose, such as guiding a thermal ablation procedure or evaluating the immediate post‐ablation response. This rate can be affected by various factors, including patient characteristics, the location and size of the tumor, and the quality of the imaging modalities used. A high applicable rate indicates that the FI technique can be effectively employed in a wide range of cases and is considered a clinically useful tool.

One study found the applicable rate of US‐CEUS to be 61.1% and that of CT/MRI‐CEUS to be 98.7%.^45^ Another study reported the applicable rates for CT/MR‐CEUS (94.8%), 3DUS‐CEUS (94.1%) and CEUS (96.0%).^50^

*Technical success rate* refers to the proportion of cases in which the desired outcome or goal is achieved using the fusion imaging technique. In the context of thermal ablation for liver cancer, technical success often means complete tumor destruction with an adequate ablative margin. Factors affecting the technical success rate may include the operator's skill and experience, the quality and accuracy of the imaging system, and the complexity of the tumor or its location. A high technical success rate indicates that the FI technique is effective in achieving the desired therapeutic outcomes and can contribute to better patient outcomes.

Seventeen studies reported the immediate technical success post‐ablation in liver neoplasms. CT/MR‐US/CEUS, US/CEUS FI, MRI‐CT FI achieved a technical success rate ranging from 81.3% to 100%,^33^, ^45^ 91.7% to 100%,^51^, ^54^ and 89%,^11^ respectively, post‐ablation in HCC patients. One study reported an immediate technical success rate of 100% using US‐US overlay in liver metastases. A non‐rigid registration approach was used in conjunction with a side‐by‐side visual comparison in one study. Five studies did not report any metric for the technical effectiveness of the intervention.

*Registration success rate* refers to the proportion of cases in which the fusion imaging software successfully aligns, or registers, the images from different imaging modalities (e.g., CT, MRI, or ultrasound). Image registration is a crucial step in FI procedures, as it enables the combination of information from multiple imaging sources to create a more comprehensive and accurate representation of the patient's anatomy and the targeted lesion. Factors that can affect the registration success rate include the quality and resolution of the images, the software's algorithm and capabilities, and the complexity of the anatomical structures being registered. A high registration success rate indicates that the FI system is effective in aligning images from different modalities, providing an accurate and reliable representation of the patient's anatomy for clinical decision‐making and interventions.

One study reported the registration success rate (indicating that the FI system is effective in aligning images from different modalities, providing an accurate and reliable representation of the patient's anatomy for clinical decision‐making and interventions). Xu et al. 2018 reported registration rates for US‐CEUS (93.8%) and CT/MRI‐CEUS (81.3%).^45^

*Fusion time* refers to the duration it takes for the FI system to align and combine images from different modalities. A shorter fusion time is desirable because it reduces the overall procedure time and allows for faster clinical decision‐making. Minimizing fusion time can also reduce the potential for patient discomfort and minimize the time spent under anesthesia or sedation, if applicable.

The median time needed to merge pre‐ and post‐ablative scans were measured in fourteen studies. The average fusion time ranged from 2 min for US‐US FI^58^ to 15 min for CT–CT fusion.^30^

*Ablative margin* is the distance between the edge of the ablated zone (treated area) and the boundary of the tumor. A sufficient AM is crucial for reducing the likelihood of local tumor progression (LTP) and ensuring the treatment's long‐term success. Studies have suggested that an AM of at least 5 mm is associated with a lower risk of LTP.

Assessment of minimal ablative safety margins was done in sixteen studies. Among the included studies, AM >5 mm was considered a cutoff for achieving adequate ablation. Quantitative 3D assessment of AM immediately post‐ablation was reported in five studies involving CT–CT fusion and four studies with US FI.^30^, ^48^, ^51^, ^56^, ^58^ Two studies compared the clinical impact of image fusion software with conventional visual comparison for the assessment of AM.^11^, ^53^

*Safety margin achieved* is the buffer zone around the tumor that includes both the tumor and the ablative margin. Achieving a safety margin ensures that the ablation procedure has effectively targeted the entire tumor while minimizing damage to the surrounding healthy tissue. A safety margin is essential for reducing complications, improving patient outcomes, and minimizing the risk of tumor recurrence.

Fifteen of the 22 studies reported the safety margin achieved for their studies. The safety margin reported in these fifteen studies ranged from 19% to 97%. Of the fifteen studies which reported their safety margins, 7/15 reported a safety margin of greater than 80%.

*Supplementary ablation carried out*: In cases where the initial ablation procedure is not sufficient to achieve a complete treatment response or the desired ablative margin, supplementary ablation may be carried out. The rate of supplementary ablation provides insight into the effectiveness of the initial ablation procedure and the need for additional treatment. A lower rate of supplementary ablation indicates that the initial treatment was more successful in achieving the desired ablative margin and safety margin. Additionally, reducing the need for supplementary ablation can help minimize patient discomfort, recovery time, and healthcare costs.

In nine studies, supplementary ablation was done following immediate evaluation by FI.^15^, ^33^, ^45^, ^46^, ^49^, ^50^, ^51^, ^58^, ^59^

*Postoperative complications*: Evaluating postoperative complications is important in assessing the overall safety and efficacy of the thermal ablation procedure using FI strategies. Complications can include infection, bleeding, damage to nearby organs or tissues, or other adverse events. Monitoring and minimizing postoperative complications is essential for ensuring patient safety, improving patient outcomes, and optimizing the success of the treatment.

Two studies reported that no major complications were reported.^40^, ^41^ Three studies reported recording major complications related to thermal ablation but did not expand on them.^33^, ^49^, ^50^ Three studies quantified the rate of major complications, ranging from 0.84% to 1.9%.^50^, ^57^, ^59^

*Local tumor progression (LTP) rate* is an important measure of the efficacy of a fusion imaging (FI) strategy in thermal ablation, as it directly reflects the success of the treatment in controlling tumor growth. A low LTP rate indicates that the ablation has effectively destroyed the targeted tumor and minimized the chances of regrowth or recurrence at the treated site. A precise and well‐performed thermal ablation should create an ablation zone that encompasses the entire tumor, with a margin of surrounding healthy tissue, to ensure complete destruction, and prevent local recurrence. FI strategies, by offering improved visualization and targeting of tumors, can potentially increase the accuracy of ablation procedures and reduce LTP rates.

In seventeen studies, LTP rates were reported during the follow‐up period. Reported LTP ranged from none (0%) to 30.3%. Most of the studies (15/17) reported LTP lower than 12% during the duration of follow‐up.

All the patient outcomes reported above are summarized in Table 6.

## 4 DISCUSSION

In published literature, previous studies have demonstrated the clinical utility of image fusion in various aspects of thermal ablation for liver neoplasms such as treatment planning, surgical guidance, and monitoring treatment efficacy. However, to the best of our knowledge, a systematic review focused on the viability and efficacy of FI systems for evaluating immediate post‐ablation response in liver neoplasms has not been reported. Thus, this is an unmet clinical need.

In this review, we assessed the efficacy and safety of different FI modalities for guiding percutaneous ablation in the treatment of liver cancer to address this unmet need. The originality of the current study lies in its focus on clinical application of anatomical image fusion techniques in evaluating the immediate therapeutic response post thermal ablation in malignant liver neoplasms. The inclusion of a diverse range of liver neoplasms in the studies reviewed adds to the strength and generalizability of the findings. The assertion of the viability of software‐based evaluation adds further support to the potential clinical utility of FI systems for evaluating immediate post‐ablation response in liver neoplasms. By identifying the capabilities, accuracy, and limitations of these FI techniques, the study findings can help to guide clinicians in selecting the most appropriate FI for immediate evaluation ultimately improving the therapeutic efficacy of the procedure, leading to better patient outcomes.

The studies included in this analysis employed various fusion imaging techniques, such as CT/MR‐CEUS, US‐CEUS, 3DUS‐CEUS, and CT/MR‐US, among others. These modalities were utilized to achieve optimal safety margins and technical success rates during percutaneous ablation procedures. The results from the included studies demonstrate that the use of fusion imaging modalities in percutaneous ablation generally leads to high technical success rates and low local tumor progression (LTP) rates.

The technical success rate refers to the proportion of cases in which the desired outcome or goal is achieved using the fusion imaging technique. In the context of thermal ablation for liver cancer, technical success often means complete tumor destruction with an adequate ablative margin. Factors affecting the technical success rate may include the operator's skill and experience, the quality and accuracy of the imaging system, and the complexity of the tumor or its location. Overall, findings showed that the immediate technical success rate using image fusion ranged from 80.7% to 100%.^45^, ^57^ In many studies, the technical success rate was above 90%. Xu et al. observed that with immediate intra‐operative evaluation through CT/MR‐CEUS FI, an adequate ablation zone could be attained in HCC patients.^45^ Additionally, LTP rates were generally low, often ranging from 0% to 14.8%.

A high registration success rate indicates that the FI system is effective in aligning images from different modalities, providing an accurate and reliable representation of the patient's anatomy for clinical decision‐making and interventions.^22^ Studies have found that FI can ensure accurate assessment of the ablation zone without prolonging the duration of ablation.^50^, ^56^ Excellent registration accuracy and short interpretation time make FI clinically relevant for determining the ablation zone immediately after ablation and guide clinicians in determining the need for additional ablation procedures. Sanga et al. achieved a concordance rate of 88% between US/CEUS intraprocedural FI and CEMRI/CECT conducted 1‐month post‐RFA.^58^ This suggests that FI techniques might be a feasible and potential intraprocedural tool for determining therapeutic effect post‐ablation in malignant liver cancer. However, only 62% of the success rate of registration was stated in the publications.

While majority of studies focused on HCC, few studies also investigated the utility of FI in the immediate evaluation of secondary liver cancers post‐ablation. These studies showed promising results in improving treatment outcomes and accurately determining the immediate therapeutic response in liver metastases.^23^, ^40^, ^41^, ^52^, ^54^ Minami et al.^62^ reported a technical success rate of 100% using US‐US overlay immediately post RFA in liver metastases. This indicates that FI can accurately determine the immediate therapeutic response in both primary and secondary liver cancer patients. However, more studies are warranted to strengthen the findings, especially for liver metastases. All the included studies employed CECT/CEMRI imaging as the reference standard for evaluating technical efficacy during the first follow‐up following ablation. The technical efficacy rates of the selected studies ranged from 89.3% to 100%.^54^, ^56^

### 4.1 FI versus single modality

FI demonstrated comparability with other imaging techniques in the immediate assessment of the ablation zone.^33^, ^49^ Ma et al. observed that CT/MR‐CEUS FI group (100%) had a higher degree of technical efficacy than the US group (86.7%).^33^ Ju et al. obtained similar outcomes with CEUS‐CT/MR FI (99.2%) compared to standard CEUS (94.2%).^49^ In the conventional US, some ablation zones appear less conspicuous due to poor spatial resolution, echoic changes following ablation, liver cirrhosis background, and adipose infiltration. CEUS, on the other hand, offers a crisp image of the ablation zone owing to a greater signal‐to‐noise ratio but cannot assess the whole ablation zone as the arterial phase only lasts a few seconds after contrast agent administration. Thus, image fusion tends to be a more reliable and objective method than routine US/CEUS for assessing the ablation zone immediately post‐ablation.

### 4.2 Multimodality FI versus monomodal FI

CT has been widely adopted by many centers due to its superior spatial and temporal resolution.^65^ Registering the preoperative and postoperative CT may help visualize pertinent anatomic features such as the tumor, ablated zone, peri‐tumoral vasculature, and peri‐ablated hyperemia. Also, the brightness, contrast, and color of the two fused images need not be adjusted.^60^ However, in many institutions, there is increasing usage of liver MR, which makes pre‐RFA CT less often needed.^11^ MR imaging is more sensitive than CT and is often used to evaluate treatment responses of liver lesions.^66^ Although CT–CT and CT‐MR FI are capable of precisely evaluating the AM, they can neither evaluate the dynamic changes nor can be performed easily during US‐guided ablation procedures.^11^, ^60^ Since the US is widely used as a guidance and monitoring tool during liver ablation, it is expected that US FI will be a more appropriate method for accurately estimating posttherapeutic response. CT/MR‐CEUS FI could be the preferred technique for assessing immediate therapeutic response to inconspicuous lesions on B‐mode US.^45^, ^50^ CEUS/US FI may be the method of choice for distinguishable lesions in B mode owing to its ease and greater registration rate.^46^, ^51^ It is simpler to align and maintain consistency between pre‐ablation and post‐ablation US images using US‐CEUS FI because US images are taken intraoperatively before ablation. US‐US FI and US‐US overlay FI enable for simultaneous assessment of AM, as the fused tumor is projected on the ablative hyperechoic zone in a US‐US overlay FI.^46^, ^54^ Compared to CT/MRI‐CEUS FI, US‐CEUS FI is less expensive, requires minimal CT/MRI resources, radiation‐free, and easier to perform in the operating room.^45^, ^50^, ^56^

### 4.3 Ablative margin

For ablation to be deemed successful, a wide ablation zone (encompassing the tumor with AM) is necessary to prevent LTP. Studies have confirmed that achieving an AM >5 mm could be considered technically successful due to the low likelihood of LTP.^15^, ^60^ A study analyzing technical effectiveness in HCC patients who had srRFA revealed a 30% decrease in relative LTP risk for every millimetric increase in the AM.^67^ Routinely employed imaging techniques, including CECT, CEMR, and CEUS cannot accurately determine whether adequate AM has been achieved.^68^ However, due to the development of image fusion, AM assessment is possible immediately following ablation. The LTP rates of malignant liver neoplasms were revealed to be reduced when pre/post images were registered immediately after ablation for measuring AM.^11^, ^60^, ^62^ A study found that the use of US‐US overlay fusion during RFA resulted in a higher rate of ablative safety margins and a lower rate of LTP compared to a US control group in HCC patients.^62^ Minami et al. reported that the use of US‐US FI overlay improved complete ablation rates in patients with liver metastases and observed no LTP during the follow‐up period. The clear visualization of the AM using US‐US FI overlay likely contributed to the improved outcomes in liver metastases.^62^

Few studies have supported the superior accuracy of quantitative AM assessment over visual AM assessment.^11^, ^53^ Quantitative assessment results in reduced errors and objective evaluation of immediate treatment response when compared to visual assessment. The clinician's ability to compare the AM side by side with the ablative zone is hampered by the uneven form of the ablative zone. On the other hand, registration software provides a better clinical picture of the ablative zone by synchronizing pre‐and post‐ablation images.^11^, ^30^, ^60^ A significant concordance rate between intraoperative CEUS derived from FI and side‐by‐side CT/MR assessments was also found by Makino et al. demonstrating that FI is reliable in the intraoperative evaluation of AM.^53^

It has been acknowledged that the ability to display spatial relationships makes three‐dimensional(3D) imaging more valuable than 2D imaging. Through the 3D technique, the ablated zone and the index tumor can be viewed from all angles.^30^, ^48^ This technique can be adapted to volumetric software that can calculate the AM automatically, thereby assisting clinicians in determining the next step. Laimer et al. found that non‐rigid registration software enables accurate assessment of safety margin volume through image fusion of pre‐ and post‐CT scans in CRLM patients undergoing sRFA. Achieving a 100% 3D safety margin of 3 mm or at least a 90% 3D safety margin of 6 mm can predict local treatment success in CRLM patients.^23^

In lesions with inadequate AM, patients should undergo immediate retreatment.^56^ Ping Ma et al. reported that intraprocedural CEUS‐CT/MR FI detected 14.5% of the HCC lesions as incompletely ablated, and following immediate supplementary ablation, all lesions achieved a 100% technical success rate.^33^ Long et al. provides evidence that immediate evaluation with CT/MR‐CEUS FI can be a useful tool in detecting incomplete ablation in difficult cases where tumors were located close to critical structures or unclear in B‐mode ultrasound.^52^ As a result, FI was able to accurately identify the need for additional ablation, leading to a higher percentage of supplemental ablation compared in difficult cases (52). Therefore, immediate evaluation using FI during ablation is a reliable way to determine the need for additional ablation for high‐risk tumors. This greatly reduces the incidence of secondary procedures, the mental and physical strain, and the medical expenditures associated with the ablation procedure.

However, adequate AM equivalent to 5 mm cannot always be achieved in all the nodules due to various factors like organ and vessel proximity, heat sink effect of the vessel, and difficulty of electrode placement while targeting large tumors.^60^ Auxiliary therapies have been used to address such problems to maximize the therapeutic effect.^46^, ^59^ In order to lessen the heat sink impact due to reduced arterial blood flow in the liver, chemoembolization pretreatment may be used. At the same time, adjuvant therapy with ethanol injection can further lower local LTP rates since it reduces blood flow around the tumor by augmenting the ablation zone. However, in challenging cases where AM cannot be achieved, the ablated zone must be evaluated cautiously during the subsequent follow‐up period.

### 4.4 Clinical implications

As described in the Introduction section, achieving a tumor‐free margin and complete eradication of microscopic invasion around the pathological periphery while maintaining liver function is essential for favorable outcomes. The evolution of tissue changes at the ablative site over time can make it challenging to accurately assess the treatment outcome, and delayed assessment can result in peripheral regrowth and hinder retreatment due to limited access.

As of current practice, the best‐case clinical scenario is having an AM of 5 mm plus signal void/zero enhancement on the subtracted imaging sequences, with no diffusion restriction within the tumor bed. This approach might help recognize sub‐total ablation and the necessity for immediate retreatment or auxillary procedures without time delay, thereby enhancing the therapeutic efficacy. Considering these challenging factors immediate accurate evaluation of the ablative zone by image fusion techniques will be helpful in achieving more favorable outcomes. FI techniques can aid in the early detection of the residual tumor and improve AM intraoperatively by displaying the spatial relationship between the ablative zone and index tumor. This can help clinicians determine whether an extension of the ablation zone is necessary during the same session, potentially improving the chances of successful treatment.

Based on the discussion above, the authors believe the clinical implications of the findings of this study on image fusion (FI) systems for evaluating the immediate post‐ablation response in liver neoplasms to be the following:

- Improved treatment planning and intervention: The study findings highlight the potential clinical utility of FI systems in various aspects of thermal ablation for liver neoplasms, including treatment planning, surgical guidance, and monitoring treatment efficacy.
- High technical success rate: Fusion of pre‐ and post‐ablation scans, in conjunction with an AM assessment immediately after the procedure, can minimize errors and objectively evaluate the procedure's technical success. We found that the included studies reported immediate technical success rate using FI ranging from 80.7% to 100%. This indicates that FI can accurately determine the immediate therapeutic response in both primary and secondary liver cancer patients, improving treatment outcomes. Moreover, FI can be particularly helpful in treatment evaluation of high‐risk tumors located near critical structures or in difficult‐to‐reach locations. This can help plan subsequent treatment and prevent injury to extrahepatic structures while increasing the operator's confidence in performing the procedure.
- Superior accuracy and efficiency: The findings of the included studies indicate FI demonstrated comparability or even superiority to single‐modality imaging techniques in the immediate assessment of the ablation zone. This suggests that FI can be a more reliable and objective method for assessing the ablation zone immediately post‐ablation.
- Versatility and cost‐effectiveness: Different types of FI systems, such as CT/MR‐CEUS FI, US‐CEUS FI, and US‐US FI, can be used depending on the specific clinical situation. This allows for tailored assessments and improved outcomes, while some FI systems (e.g., US‐CEUS FI) offer the benefits of lower cost, minimal CT/MRI resources, and no radiation exposure.
- Enhanced ablative margin (AM) assessment: Since FI enables accurate and immediate AM assessment, reducing the risk of local tumor progression (LTP), this can lead to better treatment success rates and improved patient outcomes.
- Informed decision‐making: FI can accurately identify the need for additional ablation, leading to a higher percentage of supplemental ablation in difficult cases. This will help reduce the incidence of secondary procedures, lowering the associated physical, mental, and financial burden on patients.
- Potential for auxiliary therapies: In challenging cases where adequate AM cannot be achieved, use of FI can indicate use of auxiliary therapies like chemoembolization pretreatment and adjuvant therapy with ethanol injection to maximize therapeutic effects in the short‐term.

### 4.5 Limitations

This study is limited by the concurrent nature of research and clinical practice. First, the included studies were heterogeneous in terms of patient populations, tumor types, and specific FI modalities. This heterogeneity may impact the generalizability of the results. Given the differences in biological characteristics and the potential differences in thermal ablation effect between primary and secondary liver cancers, it might be valuable to analyze them individually or to compare the differences between them as part of a future study with an appropriate sample size. Future research should aim to conduct more comprehensive and standardized studies to further validate the efficacy and safety of FI‐guided thermal ablation treatments. Second, although the registration times for FI modalities were generally short, there is still room for improvement. Future advancements in imaging technology could further enhance the efficiency and accuracy of these techniques, leading to even better clinical outcomes. Third, the long‐term outcomes of FI‐guided thermal ablation treatments were not extensively investigated in the included studies. Future research should focus on assessing the long‐term effectiveness and safety of these treatments in order to establish their role in the management of various tumor types. Fourth, there is a scarcity of prospective literature reported on the immediate posttherapeutic assessment using FI techniques that directly impacted our synthesis. Therefore, future studies should focus on conducting high‐quality RCT in this field. Fifth, there is a lack of consensus and standardization among clinicians regarding various FI techniques used for assessing AM immediately after ablation. For immediate post‐ablation therapy results, there have been just a few publications comparing the various fusion imaging methods available. Clinicians also lack agreement on a standardized protocol for analyzing AM using the same registration software. Finally, the current review omitted the gray literature, which mainly publishes the most recent updated novel methods. Finally, despite technological progress, organ movement induced by changes in posture or breathing pose problems and technical constraints for fusion registration. These difficulties include (a) different image acquisition processes resulting in different intensity distributions that do not reflect the same anatomical structures and (b) the temporal and spatial variations caused during intra‐procedural imaging.

### 4.6 Future implications

As mentioned above, the potential benefits of using FI techniques in improving short‐term outcomes and reducing the need for additional treatments make it a promising area for future research and development.

Future research must primarily focus on addressing the technical limitations (temporal and spatial variabilities and deformation) of current image fusion algorithms to achieve widespread clinical acceptance. Further improvements in the existing algorithms in terms of faster co‐registration and accuracy may also enhance the FI application. Anatomical differences in the preoperative imaging collection may be addressed to increase the accuracy of the technical fusion process, allowing for a larger range of therapeutic applications.

Image navigation systems should include registration software capabilities to offer quick quantitative data on ablation completion and treatment effectiveness. Non‐rigid co‐registration algorithms tend to have more degrees of freedom, allowing scans to better align with each other. Hence, inexperienced radiologists can benefit from utilizing non‐rigid registration algorithms for evaluating AMs and precise risk stratification for LTPs. This research also highlights the necessity to improve breathing synchronization devices and automated vessel detection and registration. Incorporating multi‐modality, multi‐temporality, parameter sensitivity, and domain specificity might simplify the development of sophisticated image processing algorithms for segmentation,^69^, ^70^, ^71^, ^72^ non‐rigid and rigid co‐registration,^29^, ^73^, ^74^ and volumetric assessment in intraprocedural quantitative AM analysis.

There is a need for further clinical trials to validate the effectiveness of commercially available co‐registration software (Ablation‐fit™,^65^, ^75^ etc.) in determining intraprocedural AM for a wider group of ablation techniques (laser ablation, cryoablation, etc.) and tumor types (subphrenic tumors, subcapsular tumors, etc.). Further research is needed to assess the clinical utility of FI on long‐term outcomes (overall survival, disease‐free survival, progression‐free survival, recurrence rates, and patients' quality of life) as well as to optimize its use in routine clinical practice.

## 5 CONCLUSION

This systematic review demonstrates the potential benefits of fusion imaging (FI) modalities in guiding thermal ablation treatments for various tumor types. The results suggest that the use of FI can enhance the technical success, safety, and local tumor progression (LTP) rates in comparison to conventional image‐guided techniques.

Fusion imaging modalities, including CT/US, MRI/US, and PET/CT, provide improved visualization and targeting of tumors, thereby increasing the precision of ablation procedures and minimizing the risk of complications. Furthermore, the low supplementary ablation rates indicate that FI‐guided treatments often achieve complete tumor destruction in a single session, reducing the need for multiple interventions.

In conclusion, fusion imaging modalities offer a promising approach to enhance the technical efficacy, safety margins, and LTP rates for thermal ablation treatments. While the current evidence supports their use in clinical practice, continued research is needed to optimize and standardize these techniques and further investigate their long‐term outcomes. To corroborate our results, large‐scale randomized studies are necessary. FI technique could precisely assess the AM and guide clinicians in making decisions for additional ablation in the same session. Future work should focus on the technological improvement of FI and its clinical applicability to a broader group of tumor types and ablation procedures.

## AUTHOR CONTRIBUTIONS

**Pragati Rai:** Conceptualization (lead); formal analysis (lead); investigation (equal); writing – original draft (lead). **Mohammed Yusuf Ansari:** Formal analysis (lead); investigation (supporting); methodology (supporting); writing – original draft (supporting); writing – review and editing (supporting). **Mohamed Warfa:** Formal analysis (supporting); investigation (supporting). **Hammad Al‐Hamar:** Data curation (lead); writing – review and editing (supporting). **Julien Abinahed:** Writing – review and editing (supporting). **Ali Barah:** Supervision (equal); writing – review and editing (supporting). **Sarada Prasad Dakua:** Conceptualization (equal); funding acquisition (lead); supervision (equal); writing – review and editing (equal). **Shidin Balakrishnan:** Conceptualization (equal); methodology (supporting); project administration (lead); supervision (equal); writing – review and editing (lead).

## FUNDING INFORMATION

This publication was made possible by NPRP‐11S‐1219‐170106 from the Qatar National Research Fund (a member of Qatar Foundation). The Open Access funding has been provided by the Qatar National Library. All opinions, findings, conclusions, or recommendations expressed in this work are those of the authors and do not necessarily reflect the views of our sponsors.

## CONFLICT OF INTEREST STATEMENT

The authors declare that the research was conducted in the absence of any commercial or financial relationships that could be construed as a potential conflict of interest.

## ETHICS STATEMENT

Not applicable.

## HUMAN SUBJECTS INFORMED CONSENT

Not applicable.

## Supporting information


Data S1.

Data S2.

Data S3.

## Data Availability

The data presented in the study have been detailed in the article/supplementary material. Further inquiries can be directed to the corresponding author.
