# Supplementary material for: Efficacy of fusion imaging for immediate post‐ablation assessment of malignant liver neoplasms: A systematic review
Source: Cancer Med. 2023 May 16;12(13):14225–51. doi: 10.1002/cam4.6089 (PMC10358230; doi:10.1002/cam4.6089)
Supplement: Supplementary file 2 — Data S2. [file CAM4-12-14225-s003.pdf]

MODIFIED NEWCASTLE - OTTAWA QUALITY ASSESSMENT SCALE

Xu et al. 2021 Retrospective

No.	Criterion	Decision rule	Score (*=1, no*=0)	Location in text
**SELECTION**				
1	Representativeness of the exposed cohort	a) Consecutive eligible participants were selected, participants were randomly selected, or all participants were invited to participate from the source population*<br>b) Not satisfying requirements in part (a), or not stated.	1	
2	Selection of the non-exposed cohort	a) Selected from the same source population*<br>b) Selected from a different source population<br>c) No description	1	
3	Ascertainment of exposure	a) Structured injury data (e.g. record completed by medical staff)*<br>b) Structured interview*<br>c) Written self-report<br>d) No description	1	
4	Demonstration that outcome of interest was not present at the start of the study	a) Yes*<br>b) No or not explicitly stated	0	
**COMPARABILITY**				
1	Comparability of cohorts on the basis of the design or analysis	a) Study controls for previous injury*<br>b) Study controls for age*<br><br>*Note:* Exposed and non-exposed individuals must be matched in the design and/or confounders must be adjusted for in the analysis. Alone statements of no differences between groups or that differences were not statistically significant are not sufficient.	0	
**OUTCOME**				
1	Assessment of outcome	a. Independent or blind assessment stated, or confirmation of the outcome by reference to secure records (e.g. imaging, structured injury data, etc.)*<br>b. record linkage (e.g. identified through ICD codes on database records)*<br>c. Self-report with no reference to original structured injury data or imaging<br>d. No description	1	
2	Was follow-up long enough for outcomes to occur?	a) Yes (≥3 months)*<br>b) No (<3 months)	1	
3	Adequacy of follow up of cohorts	a) Complete follow up – all participants accounted for*<br>b) Subjects lost to follow up unlikely to introduce bias (<15% lost to follow up, or description provided of those lost*)<br>c) Follow up rate <85% and no description of those lost provided<br>d) No statement	1	
		**SCORE:**	**6**	

MODIFIED NEWCASTLE - OTTAWA QUALITY ASSESSMENT SCALE

Xu et al 2018 Prospective

No.	Criterion	Decision rule	Score (*=1, no*=0)	Location in text
**SELECTION**				
1	Representativeness of the exposed cohort	a) Consecutive eligible participants were selected, participants were randomly selected, or all participants were invited to participate from the source population*<br>b)Not satisfying requirements in part (a), or not stated.	1	
2	Selection of the non-exposed cohort	a) Selected from the same source population*<br>b) Selected from a different source population<br>c) No description	0	
3	Ascertainment of exposure	a) Structured injury data (e.g. record completed by medical staff)*<br>b) Structured interview*<br>c) Written self-report<br>d) No description	1	
4	Demonstration that outcome of interest was not present at the start of the study	a) Yes*<br>b) No or not explicitly stated	1	
**COMPARABILITY**				
1	Comparability of cohorts on the basis of the design or analysis	a) Study controls for previous injury*<br>b) Study controls for age*<br><br>*Note:* Exposed and non-exposed individuals must be matched in the design and/or confounders must be adjusted for in the analysis. Alone statements of no differences between groups or that differences were not statistically significant are not sufficient.	0	
**OUTCOME**				
1	Assessment of outcome	a) Independent or blind assessment stated, or confirmation of the outcome by reference to secure records (e.g. imaging, structured injury data, etc.)*<br>b) record linkage (e.g. identified through ICD codes on database records)*<br>c) Self-report with no reference to original structured injury data or imaging<br>d) No description	1	
2	Was follow-up long enough for outcomes to occur?	a) Yes (≥3 months)*<br>b) No (<3 months)	0	
3	Adequacy of follow up of cohorts	a) Complete follow up – all participants accounted for*<br>b) Subjects lost to follow up unlikely to introduce bias (<15% lost to follow up, or description provided of those lost*)<br>c) Follow up rate <85% and no description of those lost provided<br>d) No statement	0	
		**SCORE:**	**4**	

MODIFIED NEWCASTLE - OTTAWA QUALITY ASSESSMENT SCALE

Xu et al 2018 Prospective

No.	Criterion	Decision rule	Score (*=1, no*=0)	Location in text
**SELECTION**				
1	Representativeness of the exposed cohort	a) Consecutive eligible participants were selected, participants were randomly selected, or all participants were invited to participate from the source population*<br>b) Not satisfying requirements in part (a), or not stated.	0	
2	Selection of the non-exposed cohort	a. Selected from the same source population*<br>b. Selected from a different source population<br>c. No description	0	
3	Ascertainment of exposure	a. Structured injury data (e.g. record completed by medical staff)*<br>b. Structured interview*<br>c. Written self-report<br>d. No description	1	
4	Demonstration that outcome of interest was not present at the start of the study	a) Yes*<br>b) No or not explicitly stated	1	
**COMPARABILITY**				
1	Comparability of cohorts on the basis of the design or analysis	a) Study controls for previous injury*<br>b) Study controls for age*<br><br>*Note:* Exposed and non-exposed individuals must be matched in the design and/or confounders must be adjusted for in the analysis. Alone statements of no differences between groups or that differences were not statistically significant are not sufficient.	0	
**OUTCOME**				
1	Assessment of outcome	a. Independent or blind assessment stated, or confirmation of the outcome by reference to secure records (e.g. imaging, structured injury data, etc.)*<br>b. record linkage (e.g. identified through ICD codes on database records)*<br>c. Self-report with no reference to original structured injury data or imaging<br>d. No description	1	
2	Was follow-up long enough for outcomes to occur?	a) Yes (≥3 months)*<br>b) No (<3 months)	0	
3	Adequacy of follow up of cohorts	a) Complete follow up – all participants accounted for*<br>b) Subjects lost to follow up unlikely to introduce bias (<15% lost to follow up, or description provided of those lost*)<br>c) Follow up rate <85% and no description of those lost provided<br>d) No statement	0	
		**SCORE:**	**4**	

MODIFIED NEWCASTLE - OTTAWA QUALITY ASSESSMENT SCALE

Ding et al 2021 Prospective

No.	Criterion	Decision rule	Score (*=1, no*=0)	Location in text
**SELECTION**				
1	Representativeness of the exposed cohort	a) Consecutive eligible participants were selected, participants were randomly selected, or all participants were invited to participate from the source population*<br>b) Not satisfying requirements in part (a), or not stated.	1	
2	Selection of the non-exposed cohort	a) Selected from the same source population*<br>b) Selected from a different source population<br>c) No description	0	
3	Ascertainment of exposure	a) Structured injury data (e.g. record completed by medical staff)*<br>b) Structured interview*<br>c) Written self-report<br>d) No description	1	
4	Demonstration that outcome of interest was not present at the start of the study	a) Yes*<br>b) No or not explicitly stated	1	
**COMPARABILITY**				
1	Comparability of cohorts on the basis of the design or analysis	a) Study controls for previous injury*<br>b) Study controls for age*<br><br>*Note:* Exposed and non-exposed individuals must be matched in the design and/or confounders must be adjusted for in the analysis. Alone statements of no differences between groups or that differences were not statistically significant are not sufficient.	0	
**OUTCOME**				
1	Assessment of outcome	a. Independent or blind assessment stated, or confirmation of the outcome by reference to secure records (e.g. imaging, structured injury data, etc.)*<br>b. record linkage (e.g. identified through ICD codes on database records)*<br>c. Self-report with no reference to original structured injury data or imaging<br>d. No description	1	
2	Was follow-up long enough for outcomes to occur?	a) Yes (≥3 months)*<br>b) No (<3 months)	0	
3	Adequacy of follow up of cohorts	a) Complete follow up – all participants accounted for*<br>b) Subjects lost to follow up unlikely to introduce bias (<15% lost to follow up, or description provided of those lost*)<br>c) Follow up rate <85% and no description of those lost provided<br>d) No statement	0	
		**SCORE:**	**4**	

MODIFIED NEWCASTLE - OTTAWA QUALITY ASSESSMENT SCALE

Li et al 2017 Prospective

No.	Criterion	Decision rule	Score (*=1, no*=0)	Location in text
**SELECTION**				
1	Representativeness of the exposed cohort	a) Consecutive eligible participants were selected, participants were randomly selected, or all participants were invited to participate from the source population*<br>b) Not satisfying requirements in part (a), or not stated.	1	
2	Selection of the non-exposed cohort	a) Selected from the same source population*<br>b) Selected from a different source population<br>c) No description	0	
3	Ascertainment of exposure	a) Structured injury data (e.g. record completed by medical staff)*<br>b) Structured interview*<br>c) Written self-report<br>d) No description	1	
4	Demonstration that outcome of interest was not present at the start of the study	a) Yes*<br>b) No or not explicitly stated	1	
**COMPARABILITY**				
1	Comparability of cohorts on the basis of the design or analysis	a) Study controls for previous injury*<br>b) Study controls for age*<br><br>*Note:* Exposed and non-exposed individuals must be matched in the design and/or confounders must be adjusted for in the analysis. Alone statements of no differences between groups or that differences were not statistically significant are not sufficient.	0	
**OUTCOME**				
1	Assessment of outcome	a. Independent or blind assessment stated, or confirmation of the outcome by reference to secure records (e.g. imaging, structured injury data, etc.)*<br>b. record linkage (e.g. identified through ICD codes on database records)*<br>c. Self-report with no reference to original structured injury data or imaging<br>d. No description	1	
2	Was follow-up long enough for outcomes to occur?	a) Yes (≥3 months)*<br>b) No (<3 months)	0	
3	Adequacy of follow up of cohorts	a) Complete follow up – all participants accounted for*<br>b) Subjects lost to follow up unlikely to introduce bias (<15% lost to follow up, or description provided of those lost*)<br>c) Follow up rate <85% and no description of those lost provided<br>d) No statement	0	
		**SCORE:**	**4**	

MODIFIED NEWCASTLE - OTTAWA QUALITY ASSESSMENT SCALE

Li et al 2016 Prospective

No.	Criterion	Decision rule	Score (*=1, no*=0)	Location in text
**SELECTION**				
1	Representativeness of the exposed cohort	a) Consecutive eligible participants were selected, participants were randomly selected, or all participants were invited to participate from the source population*<br>b) Not satisfying requirements in part (a), or not stated.	1	
2	Selection of the non-exposed cohort	a) Selected from the same source population*<br>b) Selected from a different source population<br>c) No description	0	
3	Ascertainment of exposure	a) Structured injury data (e.g. record completed by medical staff)*<br>b) Structured interview*<br>c) Written self-report<br>d) No description	1	
4	Demonstration that outcome of interest was not present at the start of the study	a) Yes*<br>b) No or not explicitly stated	1	
**COMPARABILITY**				
1	Comparability of cohorts on the basis of the design or analysis	a) Study controls for previous injury*<br>b) Study controls for age*<br><br>*Note:* Exposed and non-exposed individuals must be matched in the design and/or confounders must be adjusted for in the analysis. Alone statements of no differences between groups or that differences were not statistically significant are not sufficient.	0	
**OUTCOME**				
1	Assessment of outcome	a. Independent or blind assessment stated, or confirmation of the outcome by reference to secure records (e.g. imaging, structured injury data, etc.)*<br>b. record linkage (e.g. identified through ICD codes on database records)*<br>c. Self-report with no reference to original structured injury data or imaging<br>d. No description	1	
2	Was follow-up long enough for outcomes to occur?	a) Yes (≥3 months)*<br>b) No (<3 months)	0	
3	Adequacy of follow up of cohorts	a) Complete follow up – all participants accounted for*<br>b) Subjects lost to follow up unlikely to introduce bias (<15% lost to follow up, or description provided of those lost*)<br>c) Follow up rate <85% and no description of those lost provided<br>d) No statement	0	
		**SCORE:**	**4**	

MODIFIED NEWCASTLE - OTTAWA QUALITY ASSESSMENT SCALE

Zhang et al 2019 Prospective

No.	Criterion	Decision rule	Score (*=1, no*=0)	Location in text
**SELECTION**				
1	Representativeness of the exposed cohort	a) Consecutive eligible participants were selected, participants were randomly selected, or all participants were invited to participate from the source population*<br>b) Not satisfying requirements in part (a), or not stated.	1	
2	Selection of the non-exposed cohort	a) Selected from the same source population*<br>b) Selected from a different source population<br>c) No description	0	
3	Ascertainment of exposure	a) Structured injury data (e.g. record completed by medical staff)*<br>b) Structured interview*<br>c) Written self-report<br>d) No description	1	
4	Demonstration that outcome of interest was not present at the start of the study	a) Yes*<br>b) No or not explicitly stated	1	
**COMPARABILITY**				
1	Comparability of cohorts on the basis of the design or analysis	a) Study controls for previous injury*<br>b) Study controls for age*<br><br>*Note:* Exposed and non-exposed individuals must be matched in the design and/or confounders must be adjusted for in the analysis. Alone statements of no differences between groups or that differences were not statistically significant are not sufficient.	0	
**OUTCOME**				
1	Assessment of outcome	a. Independent or blind assessment stated, or confirmation of the outcome by reference to secure records (e.g. imaging, structured injury data, etc.)*<br>b. record linkage (e.g. identified through ICD codes on database records)*<br>c. Self-report with no reference to original structured injury data or imaging<br>d. No description	1	
2	Was follow-up long enough for outcomes to occur?	a) Yes (≥3 months)*<br>b) No (<3 months)	1	
3	Adequacy of follow up of cohorts	a) Complete follow up – all participants accounted for*<br>b) Subjects lost to follow up unlikely to introduce bias (<15% lost to follow up, or description provided of those lost*)<br>c) Follow up rate <85% and no description of those lost provided<br>d) No statement	1	
		**SCORE:**	**6**	

MODIFIED NEWCASTLE - OTTAWA QUALITY ASSESSMENT SCALE

Yoon et al Prospective

No.	Criterion	Decision rule	Score (*=1, no*=0)	Location in text
**SELECTION**				
1	Representativeness of the exposed cohort	a) Consecutive eligible participants were selected, participants were randomly selected, or all participants were invited to participate from the source population*<br>b) Not satisfying requirements in part (a), or not stated.	1	
2	Selection of the non-exposed cohort	a) Selected from the same source population*<br>b) Selected from a different source population<br>c) No description	0	
3	Ascertainment of exposure	a) Structured injury data (e.g. record completed by medical staff)*<br>b) Structured interview*<br>c) Written self-report<br>d) No description	1	
4	Demonstration that outcome of interest was not present at the start of the study	a) Yes*<br>b) No or not explicitly stated	1	
**COMPARABILITY**				
1	Comparability of cohorts on the basis of the design or analysis	a) Study controls for previous injury*<br>b) Study controls for age*<br><br>*Note:* Exposed and non-exposed individuals must be matched in the design and/or confounders must be adjusted for in the analysis. Alone statements of no differences between groups or that differences were not statistically significant are not sufficient.	0	
**OUTCOME**				
1	Assessment of outcome	a. Independent or blind assessment stated, or confirmation of the outcome by reference to secure records (e.g. imaging, structured injury data, etc.)*<br>b. record linkage (e.g. identified through ICD codes on database records)*<br>c. Self-report with no reference to original structured injury data or imaging<br>d. No description	1	
2	Was follow-up long enough for outcomes to occur?	a) Yes (≥3 months)*<br>b) No (<3 months)	1	
3	Adequacy of follow up of cohorts	a) Complete follow up – all participants accounted for*<br>b) Subjects lost to follow up unlikely to introduce bias (<15% lost to follow up, or description provided of those lost*)<br>c) Follow up rate <85% and no description of those lost provided<br>d) No statement	1	
		**SCORE:**	**6**	

MODIFIED NEWCASTLE - OTTAWA QUALITY ASSESSMENT SCALE

Ye et al 2019

No.	Criterion	Decision rule	Score (*=1, no*=0)	Location in text
**SELECTION**				
1	Representativeness of the exposed cohort	a) Consecutive eligible participants were selected, participants were randomly selected, or all participants were invited to participate from the source population*<br>b) Not satisfying requirements in part (a), or not stated.	1	
2	Selection of the non-exposed cohort	a) Selected from the same source population*<br>b) Selected from a different source population<br>c) No description	0	
3	Ascertainment of exposure	a) Structured injury data (e.g. record completed by medical staff)*<br>b) Structured interview*<br>c) Written self-report<br>d) No description	1	
4	Demonstration that outcome of interest was not present at the start of the study	a) Yes*<br>b) No or not explicitly stated	1	
**COMPARABILITY**				
1	Comparability of cohorts on the basis of the design or analysis	a) Study controls for previous injury*<br>b) Study controls for age*<br><br>*Note:* Exposed and non-exposed individuals must be matched in the design and/or confounders must be adjusted for in the analysis. Alone statements of no differences between groups or that differences were not statistically significant are not sufficient.	0	
**OUTCOME**				
1	Assessment of outcome	a. Independent or blind assessment stated, or confirmation of the outcome by reference to secure records (e.g. imaging, structured injury data, etc.)*<br>b. record linkage (e.g. identified through ICD codes on database records)*<br>c. Self-report with no reference to original structured injury data or imaging<br>d. No description	1	
2	Was follow-up long enough for outcomes to occur?	a) Yes (≥3 months)*<br>b) No (<3 months)	`1	
3	Adequacy of follow up of cohorts	a) Complete follow up – all participants accounted for*<br>b) Subjects lost to follow up unlikely to introduce bias (<15% lost to follow up, or description provided of those lost*)<br>c) Follow up rate <85% and no description of those lost provided<br>d) No statement	1	
		**SCORE:**	**6**	

MODIFIED NEWCASTLE - OTTAWA QUALITY ASSESSMENT SCALE

Sanga et al 2020

No.	Criterion	Decision rule	Score (*=1, no*=0)	Location in text
**SELECTION**				
1	Representativeness of the exposed cohort	a) Consecutive eligible participants were selected, participants were randomly selected, or all participants were invited to participate from the source population*<br>b) Not satisfying requirements in part (a), or not stated.	1	
2	Selection of the non-exposed cohort	a) Selected from the same source population*<br>b) Selected from a different source population<br>c) No description	0	
3	Ascertainment of exposure	a) Structured injury data (e.g. record completed by medical staff)*<br>b) Structured interview*<br>c) Written self-report<br>d) No description	1	
4	Demonstration that outcome of interest was not present at the start of the study	a) Yes*<br>b) No or not explicitly stated	1	
**COMPARABILITY**				
1	Comparability of cohorts on the basis of the design or analysis	a) Study controls for previous injury*<br>b) Study controls for age*<br><br>*Note:* Exposed and non-exposed individuals must be matched in the design and/or confounders must be adjusted for in the analysis. Alone statements of no differences between groups or that differences were not statistically significant are not sufficient.	0	
**OUTCOME**				
1	Assessment of outcome	a. Independent or blind assessment stated, or confirmation of the outcome by reference to secure records (e.g. imaging, structured injury data, etc.)*<br>b. record linkage (e.g. identified through ICD codes on database records)*<br>c. Self-report with no reference to original structured injury data or imaging<br>d. No description	1	
2	Was follow-up long enough for outcomes to occur?	a) Yes (≥3 months)*<br>b) No (<3 months)	1	
3	Adequacy of follow up of cohorts	a) Complete follow up – all participants accounted for*<br>b) Subjects lost to follow up unlikely to introduce bias (<15% lost to follow up, or description provided of those lost*)<br>c) Follow up rate <85% and no description of those lost provided<br>d) No statement	1	
		**SCORE:**	**6**	

MODIFIED NEWCASTLE - OTTAWA QUALITY ASSESSMENT SCALE

Long et al 2020

No.	Criterion	Decision rule	Score (*=1, no*=0)	Location in text
**SELECTION**				
1	Representativeness of the exposed cohort	a) Consecutive eligible participants were selected, participants were randomly selected, or all participants were invited to participate from the source population*<br>b) Not satisfying requirements in part (a), or not stated.	1	
2	Selection of the non-exposed cohort	a) Selected from the same source population*<br>b) Selected from a different source population<br>c) No description	1	
3	Ascertainment of exposure	a) Structured injury data (e.g. record completed by medical staff)*<br>b) Structured interview*<br>c) Written self-report<br>d) No description	1	
4	Demonstration that outcome of interest was not present at the start of the study	a) Yes*<br>b) No or not explicitly stated	0	
**COMPARABILITY**				
1	Comparability of cohorts on the basis of the design or analysis	a) Study controls for previous injury*<br>b) Study controls for age*<br><br>*Note:* Exposed and non-exposed individuals must be matched in the design and/or confounders must be adjusted for in the analysis. Alone statements of no differences between groups or that differences were not statistically significant are not sufficient.	0	
**OUTCOME**				
1	Assessment of outcome	a. Independent or blind assessment stated, or confirmation of the outcome by reference to secure records (e.g. imaging, structured injury data, etc.)*<br>b. record linkage (e.g. identified through ICD codes on database records)*<br>c. Self-report with no reference to original structured injury data or imaging<br>d. No description	1	
2	Was follow-up long enough for outcomes to occur?	a) Yes (≥3 months)*<br>b) No (<3 months)	1	
3	Adequacy of follow up of cohorts	a) Complete follow up – all participants accounted for*<br>b) Subjects lost to follow up unlikely to introduce bias (<15% lost to follow up, or description provided of those lost*)<br>c) Follow up rate <85% and no description of those lost provided<br>d) No statement	1	
		**SCORE:**		

MODIFIED NEWCASTLE - OTTAWA QUALITY ASSESSMENT SCALE

Ju et al 2019

No.	Criterion	Decision rule	Score (*=1, no*=0)	Location in text
**SELECTION**				
1	Representativeness of the exposed cohort	a) Consecutive eligible participants were selected, participants were randomly selected, or all participants were invited to participate from the source population*<br>b) Not satisfying requirements in part (a), or not stated.	1	
2	Selection of the non-exposed cohort	a) Selected from the same source population*<br>b) Selected from a different source population<br>c) No description	0	
3	Ascertainment of exposure	a) Structured injury data (e.g. record completed by medical staff)*<br>b) Structured interview*<br>c) Written self-report<br>d) No description	1	
4	Demonstration that outcome of interest was not present at the start of the study	a) Yes*<br>b) No or not explicitly stated	0	
**COMPARABILITY**				
1	Comparability of cohorts on the basis of the design or analysis	a) Study controls for previous injury*<br>b) Study controls for age*<br><br>*Note:* Exposed and non-exposed individuals must be matched in the design and/or confounders must be adjusted for in the analysis. Alone statements of no differences between groups or that differences were not statistically significant are not sufficient.	0	
**OUTCOME**				
1	Assessment of outcome	a. Independent or blind assessment stated, or confirmation of the outcome by reference to secure records (e.g. imaging, structured injury data, etc.)*<br>b. record linkage (e.g. identified through ICD codes on database records)*<br>c. Self-report with no reference to original structured injury data or imaging<br>d. No description	1	
2	Was follow-up long enough for outcomes to occur?	a) Yes (≥3 months)*<br>b) No (<3 months)	1	
3	Adequacy of follow up of cohorts	a) Complete follow up – all participants accounted for*<br>b) Subjects lost to follow up unlikely to introduce bias (<15% lost to follow up, or description provided of those lost*)<br>c) Follow up rate <85% and no description of those lost provided<br>d) No statement	0	
		**SCORE:**	**4**	

MODIFIED NEWCASTLE - OTTAWA QUALITY ASSESSMENT SCALE

Ping Ma et al 2019

No.	Criterion	Decision rule	Score (*=1, no*=0)	Location in text
**SELECTION**				
1	Representativeness of the exposed cohort	) Consecutive eligible participants were selected, participants were randomly selected, or all participants were invited to participate from the source population*<br>) Not satisfying requirements in part (a), or not stated.	1	
2	Selection of the non-exposed cohort	a) Selected from the same source population*<br>b) Selected from a different source population<br>c) No description	1	
3	Ascertainment of exposure	a) Structured injury data (e.g. record completed by medical staff)*<br>b) Structured interview*<br>c) Written self-report<br>d) No description	1	
4	Demonstration that outcome of interest was not present at the start of the study	a) Yes*<br>b) No or not explicitly stated	0	
**COMPARABILITY**				
1	Comparability of cohorts on the basis of the design or analysis	a) Study controls for previous injury*<br>b) Study controls for age*<br><br>*Note:* Exposed and non-exposed individuals must be matched in the design and/or confounders must be adjusted for in the analysis. Alone statements of no differences between groups or that differences were not statistically significant are not sufficient.	0	
**OUTCOME**				
1	Assessment of outcome	a. Independent or blind assessment stated, or confirmation of the outcome by reference to secure records (e.g. imaging, structured injury data, etc.)*<br>b. record linkage (e.g. identified through ICD codes on database records)*<br>c. Self-report with no reference to original structured injury data or imaging<br>d. No description	1	
2	Was follow-up long enough for outcomes to occur?	a) Yes (≥3 months)*<br>b) No (<3 months)	1	
3	Adequacy of follow up of cohorts	a) Complete follow up – all participants accounted for*<br>b) Subjects lost to follow up unlikely to introduce bias (<15% lost to follow up, or description provided of those lost*)<br>c) Follow up rate <85% and no description of those lost provided<br>d) No statement	1	
		**SCORE:**	**6**	

MODIFIED NEWCASTLE - OTTAWA QUALITY ASSESSMENT SCALE

Wang et al 2018

No.	Criterion	Decision rule	Score (*=1, no*=0)	Location in text
**SELECTION**				
1	Representativeness of the exposed cohort	a) Consecutive eligible participants were selected, participants were randomly selected, or all participants were invited to participate from the source population*<br>b) Not satisfying requirements in part (a), or not stated.	1	
2	Selection of the non-exposed cohort	a) Selected from the same source population*<br>b) Selected from a different source population<br>c) No description	0	
3	Ascertainment of exposure	a) Structured injury data (e.g. record completed by medical staff)*<br>b) Structured interview*<br>c) Written self-report<br>d) No description	1	
4	Demonstration that outcome of interest was not present at the start of the study	a) Yes*<br>b) No or not explicitly stated	0	
**COMPARABILITY**				
1	Comparability of cohorts on the basis of the design or analysis	a) Study controls for previous injury*<br>b) Study controls for age*<br><br>*Note:* Exposed and non-exposed individuals must be matched in the design and/or confounders must be adjusted for in the analysis. Alone statements of no differences between groups or that differences were not statistically significant are not sufficient.	0	
**OUTCOME**				
1	Assessment of outcome	a. Independent or blind assessment stated, or confirmation of the outcome by reference to secure records (e.g. imaging, structured injury data, etc.)*<br>b. record linkage (e.g. identified through ICD codes on database records)*<br>c. Self-report with no reference to original structured injury data or imaging<br>d. No description	1	
2	Was follow-up long enough for outcomes to occur?	a) Yes ($\geq$3 months)*<br>b) No (<3 months)	1	
3	Adequacy of follow up of cohorts	a) Complete follow up – all participants accounted for*<br>b) Subjects lost to follow up unlikely to introduce bias (<15% lost to follow up, or description provided of those lost*)<br>c) Follow up rate <85% and no description of those lost provided<br>d) No statement	1	
		**SCORE: 5**		

MODIFIED NEWCASTLE - OTTAWA QUALITY ASSESSMENT SCALE

Minami et al 2018

No.	Criterion	Decision rule	Score (*=1, no*=0)	Location in text
**SELECTION**				
1	Representativeness of the exposed cohort	a) Consecutive eligible participants were selected, participants were randomly selected, or all participants were invited to participate from the source population*<br>b) Not satisfying requirements in part (a), or not stated.	1	
2	Selection of the non-exposed cohort	a) Selected from the same source population*<br>b) Selected from a different source population<br>c) No description	0	
3	Ascertainment of exposure	a) Structured injury data (e.g. record completed by medical staff)*<br>b) Structured interview*<br>c) Written self-report<br>d) No description	1	
4	Demonstration that outcome of interest was not present at the start of the study	a) Yes*<br>b) No or not explicitly stated	0	
**COMPARABILITY**				
1	Comparability of cohorts on the basis of the design or analysis	a) Study controls for previous injury*<br>b) Study controls for age*<br><br>*Note:* Exposed and non-exposed individuals must be matched in the design and/or confounders must be adjusted for in the analysis. Alone statements of no differences between groups or that differences were not statistically significant are not sufficient.	0	
**OUTCOME**				
1	Assessment of outcome	a. Independent or blind assessment stated, or confirmation of the outcome by reference to secure records (e.g. imaging, structured injury data, etc.)*<br>b. record linkage (e.g. identified through ICD codes on database records)*<br>c. Self-report with no reference to original structured injury data or imaging<br>d. No description	1	
2	Was follow-up long enough for outcomes to occur?	a) Yes (≥3 months)*<br>b) No (<3 months)	1	
3	Adequacy of follow up of cohorts	a) Complete follow up – all participants accounted for*<br>b) Subjects lost to follow up unlikely to introduce bias (<15% lost to follow up, or description provided of those lost*)<br>c) Follow up rate <85% and no description of those lost provided<br>d) No statement	1	
		**SCORE:**	**5**	

MODIFIED NEWCASTLE - OTTAWA QUALITY ASSESSMENT SCALE

Wu et al 2018

No.	Criterion	Decision rule	Score (*=1, no*=0)	Location in text
**SELECTION**				
1	Representativeness of the exposed cohort	a) Consecutive eligible participants were selected, participants were randomly selected, or all participants were invited to participate from the source population*<br>b) Not satisfying requirements in part (a), or not stated.	1	
2	Selection of the non-exposed cohort	a) Selected from the same source population*<br>b) Selected from a different source population<br>c) No description	0	
3	Ascertainment of exposure	a) Structured injury data (e.g. record completed by medical staff)*<br>b) Structured interview*<br>c) Written self-report<br>d) No description	1	
4	Demonstration that outcome of interest was not present at the start of the study	a) Yes*<br>b) No or not explicitly stated	0	
**COMPARABILITY**				
1	Comparability of cohorts on the basis of the design or analysis	a) Study controls based on image guided intervention *<br>b) Study controls for age*<br><br>*Note:* Exposed and non-exposed individuals must be matched in the design and/or confounders must be adjusted for in the analysis. Alone statements of no differences between groups or that differences were not statistically significant are not sufficient.	0	
**OUTCOME**				
1	Assessment of outcome	a. Independent or blind assessment stated, or confirmation of the outcome by reference to secure records (e.g. imaging, structured injury data, etc.)*<br>b. record linkage (e.g. identified through ICD codes on database records)*<br>c. Self-report with no reference to original structured injury data or imaging<br>d. No description	1	
2	Was follow-up long enough for outcomes to occur?	a) Yes (≥3 months)*<br>b) No (<3 months)	1	
3	Adequacy of follow up of cohorts	a) Complete follow up – all participants accounted for*<br>b) Subjects lost to follow up unlikely to introduce bias (<15% lost to follow up, or description provided of those lost*)<br>c) Follow up rate <85% and no description of those lost provided<br>d) No statement	1	
		**SCORE:**	**5**	

MODIFIED NEWCASTLE - OTTAWA QUALITY ASSESSMENT SCALE

Makino et al 2016

No.	Criterion	Decision rule	Score (*=1, no*=0)	Location in text
**SELECTION**				
1	Representativeness of the exposed cohort	a) Consecutive eligible participants were selected, participants were randomly selected, or all participants were invited to participate from the source population*<br>b) Not satisfying requirements in part (a), or not stated.	1	
2	Selection of the non-exposed cohort	a) Selected from the same source population*<br>b) Selected from a different source population<br>c) No description	1	
3	Ascertainment of exposure	a) Structured injury data (e.g. record completed by medical staff)*<br>b) Structured interview*<br>c) Written self-report<br>d) No description	1	
4	Demonstration that outcome of interest was not present at the start of the study	a) Yes*<br>b) No or not explicitly stated	0	
**COMPARABILITY**				
1	Comparability of cohorts on the basis of the design or analysis	a) Study controls for previous injury*<br>b) Study controls for age*<br><br>*Note:* Exposed and non-exposed individuals must be matched in the design and/or confounders must be adjusted for in the analysis. Alone statements of no differences between groups or that differences were not statistically significant are not sufficient.	0	
**OUTCOME**				
1	Assessment of outcome	a. Independent or blind assessment stated, or confirmation of the outcome by reference to secure records (e.g. imaging, structured injury data, etc.)*<br>b. record linkage (e.g. identified through ICD codes on database records)*<br>c. Self-report with no reference to original structured injury data or imaging<br>d. No description	1	
2	Was follow-up long enough for outcomes to occur?	a) Yes (≥3 months)*<br>b) No (<3 months)	0	
3	Adequacy of follow up of cohorts	a) Complete follow up – all participants accounted for*<br>b) Subjects lost to follow up unlikely to introduce bias (<15% lost to follow up, or description provided of those lost*)<br>c) Follow up rate <85% and no description of those lost provided<br>d) No statement	0	
		**SCORE: 4**		

MODIFIED NEWCASTLE - OTTAWA QUALITY ASSESSMENT SCALE

Minami et al 2017

No.	Criterion	Decision rule	Score (*=1, no*=0)	Location in text
**SELECTION**				
1	Representativeness of the exposed cohort	a) Consecutive eligible participants were selected, participants were randomly selected, or all participants were invited to participate from the source population*<br>b) Not satisfying requirements in part (a), or not stated.	1	
2	Selection of the non-exposed cohort	a) Selected from the same source population*<br>b) Selected from a different source population<br>c) No description	0	
3	Ascertainment of exposure	a) Structured injury data (e.g. record completed by medical staff)*<br>b) Structured interview*<br>c) Written self-report<br>d) No description	1	
4	Demonstration that outcome of interest was not present at the start of the study	a) Yes*<br>b) No or not explicitly stated	0	
**COMPARABILITY**				
1	Comparability of cohorts on the basis of the design or analysis	a) Study controls for previous injury*<br>b) Study controls for age*<br><br>*Note:* Exposed and non-exposed individuals must be matched in the design and/or confounders must be adjusted for in the analysis. Alone statements of no differences between groups or that differences were not statistically significant are not sufficient.	0	
**OUTCOME**				
1	Assessment of outcome	a. Independent or blind assessment stated, or confirmation of the outcome by reference to secure records (e.g. imaging, structured injury data, etc.)*<br>b. record linkage (e.g. identified through ICD codes on database records)*<br>c. Self-report with no reference to original structured injury data or imaging<br>d. No description	1	
2	Was follow-up long enough for outcomes to occur?	a) Yes (≥3 months)*<br>b) No (<3 months)	1	
3	Adequacy of follow up of cohorts	a) Complete follow up – all participants accounted for*<br>b) Subjects lost to follow up unlikely to introduce bias (<15% lost to follow up, or description provided of those lost*)<br>c) Follow up rate <85% and no description of those lost provided<br>d) No statement	1	
		**SCORE: 5**		

MODIFIED NEWCASTLE - OTTAWA QUALITY ASSESSMENT SCALE

Laimer et al 2020

No.	Criterion	Decision rule	Score (*=1, no*=0)	Location in text
**SELECTION**				
1	Representativeness of the exposed cohort	a) Consecutive eligible participants were selected, participants were randomly selected, or all participants were invited to participate from the source population*<br>b) Not satisfying requirements in part (a), or not stated.	1	
2	Selection of the non-exposed cohort	a) Selected from the same source population*<br>b) Selected from a different source population<br>c) No description	0	
3	Ascertainment of exposure	a) Structured injury data (e.g. record completed by medical staff)*<br>b) Structured interview*<br>c) Written self-report<br>d) No description	1	
4	Demonstration that outcome of interest was not present at the start of the study	a) Yes*<br>b) No or not explicitly stated	0	
**c) COMPARABILITY**				
1	Comparability of cohorts on the basis of the design or analysis	a) Study controls for previous injury*<br>b) Study controls for age*<br><br>*Note:* Exposed and non-exposed individuals must be matched in the design and/or confounders must be adjusted for in the analysis. Alone statements of no differences between groups or that differences were not statistically significant are not sufficient.	0	
**c) OUTCOME**				
1	Assessment of outcome	a) Independent or blind assessment stated, or confirmation of the outcome by reference to secure records (e.g. imaging, structured injury data, etc.)*<br>b) record linkage (e.g. identified through ICD codes on database records)*<br>c) Self-report with no reference to original structured injury data or imaging<br>d) No description	1	
2	Was follow-up long enough for outcomes to occur?	a) Yes (≥3 months)*<br>b) No (<3 months)	1	
3	Adequacy of follow up of cohorts	a) Complete follow up – all participants accounted for*<br>b) Subjects lost to follow up unlikely to introduce bias (<15% lost to follow up, or description provided of those lost*)<br>c) Follow up rate <85% and no description of those lost provided<br>d) No statement	1	
		**SCORE: 5**		

MODIFIED NEWCASTLE - OTTAWA QUALITY ASSESSMENT SCALE

Laimer et al 2021

No.	Criterion	Decision rule	Score (*=1, no*=0)	Location in text
**SELECTION**				
1	Representativeness of the exposed cohort	a) Consecutive eligible participants were selected, participants were randomly selected, or all participants were invited to participate from the source population*<br>b) Not satisfying requirements in part (a), or not stated.	1	
2	Selection of the non-exposed cohort	a) Selected from the same source population*<br>b) Selected from a different source population<br>c) No description	0	
3	Ascertainment of exposure	a) Structured injury data (e.g. record completed by medical staff)*<br>b) Structured interview*<br>c) Written self-report<br>d) No description	1	
4	Demonstration that outcome of interest was not present at the start of the study	a) Yes*<br>b) No or not explicitly stated	0	
**c) COMPARABILITY**				
1	Comparability of cohorts on the basis of the design or analysis	a) Study controls for previous injury*<br>b) Study controls for age*<br><br>*Note:* Exposed and non-exposed individuals must be matched in the design and/or confounders must be adjusted for in the analysis. Alone statements of no differences between groups or that differences were not statistically significant are not sufficient.	0	
**c) OUTCOME**				
1	Assessment of outcome	a) Independent or blind assessment stated, or confirmation of the outcome by reference to secure records (e.g. imaging, structured injury data, etc.)*<br>b) record linkage (e.g. identified through ICD codes on database records)*<br>c) Self-report with no reference to original structured injury data or imaging<br>d) No description	1	
2	Was follow-up long enough for outcomes to occur?	a) Yes (≥3 months)*<br>b) No (<3 months)	1	
3	Adequacy of follow up of cohorts	a) Complete follow up – all participants accounted for*<br>b) Subjects lost to follow up unlikely to introduce bias (<15% lost to follow up, or description provided of those lost*)<br>c) Follow up rate <85% and no description of those lost provided<br>d) No statement	1	
		**SCORE:** **5**		

MODIFIED NEWCASTLE - OTTAWA QUALITY ASSESSMENT SCALE

Minami et al 2016

No.	Criterion	Decision rule	Score (*=1, no*=0)	Location in text
**SELECTION**				
1	Representativeness of the exposed cohort	a) Consecutive eligible participants were selected, participants were randomly selected, or all participants were invited to participate from the source population*<br>b) Not satisfying requirements in part (a), or not stated.	1	
2	Selection of the non-exposed cohort	a) Selected from the same source population*<br>b) Selected from a different source population<br>c)No description		
3	Ascertainment of exposure	a) Structured injury data (e.g. record completed by medical staff)*<br>b) Structured interview*<br>c) Written self-report<br>d) No description	1	
4	Demonstration that outcome of interest was not present at the start of the study	a) Yes*<br>b) No or not explicitly stated	0	
**c) COMPARABILITY**				
1	Comparability of cohorts on the basis of the design or analysis	a) Study controls for previous injury*<br>b) Study controls for age*<br><br>*Note:* Exposed and non-exposed individuals must be matched in the design and/or confounders must be adjusted for in the analysis. Alone statements of no differences between groups or that differences were not statistically significant are not sufficient.	0	
**c) OUTCOME**				
1	Assessment of outcome	a) Independent or blind assessment stated, or confirmation of the outcome by reference to secure records (e.g. imaging, structured injury data, etc.)*<br>b) record linkage (e.g. identified through ICD codes on database records)*<br>c) Self-report with no reference to original structured injury data or imaging<br>d) No description	1	
2	Was follow-up long enough for outcomes to occur?	a) Yes (≥3 months)*<br>b) No (<3 months)	1	
3	Adequacy of follow up of cohorts	a) Complete follow up – all participants accounted for*<br>b) Subjects lost to follow up unlikely to introduce bias (<15% lost to follow up, or description provided of those lost*)<br>c) Follow up rate <85% and no description of those lost provided<br>d) No statement	1	
		**SCORE:** **5**		

Risk of Bias assessment

.

Huang 2019 (RCT)

Domain	Risk of bias<br>Low	 High	 Unclear	Support for judgement<br>*(include direct quotes where available with explanatory comments)*	Location in text or source *(pg & ¶/fig/table/other)*
Random sequence generation<br>*(selection bias)*	**☐**	☐	☐	Using a computer random number generator	
Allocation concealment<br>*(selection bias)*	☐	**☐**	☐		
Blinding of participants and personnel<br>*(performance bias)*	☐	**☐**	☐	Blinding of participants and key study personnel ensured, and unlikely that the blinding could have been broken;	
(if separate judgement by outcome(s) required)	☐	☐	☐	Outcome group:	
Blinding of outcome assessment<br>*(detection bias)*	**☐**	☐	☐	Outcome group: All/	
(if separate judgement by outcome(s) required)	☐	☐	☐	Outcome group:	
Incomplete outcome data<br>*(attrition bias)*	**☐**	☐	☐	Outcome group: All/	

(if separate judgement by outcome(s) required)	☐ ☐ ☐	Outcome group:	
Selective outcome reporting?<br><br>*(reporting bias)*	☐ ☐ ☐		
Other bias	☐ ☐ ☐		
Notes:			
