# Supplementary material for: Efficacy of fusion imaging for immediate post‐ablation assessment of malignant liver neoplasms: A systematic review
Source: Cancer Med. 2023 May 16;12(13):14225–51. doi: 10.1002/cam4.6089 (PMC10358230; doi:10.1002/cam4.6089)
Supplement: Supplementary file 3 — Data S3. [file CAM4-12-14225-s002.pdf]

Embase®

Embase Session Results

No.	Query	Results
#18	**#11** AND **#16** AND [2016-2021]/py	1,728
#17	**#11** AND **#16**	4,003
#16	**#12** OR **#13** OR **#14** OR **#15**	346,602
#15	**ablative** AND **margin**	626
#14	**volumetric**	62,993
#13	**'three dimensional'**	285,820
#12	**intraprocedural**	5,536
#11	**#9** OR **#10**	36,638
#10	**fusion** AND **imaging** OR **'image fusion'**	34,967
#9	**#6** AND **#7** AND **#8**	1,691
#8	**'treatment outcome'**	909,034
#7	**#3** OR **#4** OR **#5**	112,715
#6	**#1** OR **#2**	220,703
#5	**microwave**	53,199
#4	**'radiofrequency ablation'**	43,739
#3	**'ablation therapy'**	22,643
#2	**'liver tumor'**	67,169
#1	**'liver cell carcinoma'**/exp	177,713

Embase®

Search Name: Cochrane SEARCH
Date Run: 09/08/2021 14:06:36
Comment: 2

ID	Search	Hits
#1	MeSH descriptor: [Liver Neoplasms] explode all trees	3074
#2	MeSH descriptor: [Carcinoma, Hepatocellular] explode all trees	1837
#3	#1 OR # 2	1053335
#4	MeSH descriptor: [Ablation Techniques] explode all trees	5934
#5	MeSH descriptor: [Radiofrequency Ablation] explode all trees	1571
#6	MeSH descriptor: [Microwaves] explode all trees	220
#7	#4 OR #5 OR #6	6094
#8	MeSH descriptor: [Treatment Outcome] explode all trees	145303
#9	#3 AND #7 AND #8	2163
#10	#3 AND #7 AND #8 with Cochrane Library publication date Between Jan 2016 and Jul 2021	717
#11	fusion imaging OR image fusion	1179
#12	#9 OR #11	3336
#13	#10 OR #11	1893
#14	ablative margin OR Intraprocedural OR three dimensional OR volumetric assessment	6709
#15	#12 AND #14	150
#16	#13 AND #14	122

Search nur	Query	Sort By	Filters	Search Det	Results	Time
6	(((((("carcinoma, hepatocellular"			((("carcino	2,351	4:00:44
5	((((("carcinoma, hepatocellular"[			(("carcinor	42,146	3:59:31
4	ablative margin or intraprocedur			(("ablative	247,630	3:58:32
3	Fusion imaging			("fusions"[	39,546	3:57:39
2	(((("carcinoma, hepatocellular"[I			("carcinom	2,650	3:56:00
